# NFIB–MLL1 complex is required for the stemness and Dlx5-dependent osteogenic differentiation of C3H10T1/2 mesenchymal stem cells

**DOI:** 10.1016/j.jbc.2023.105193

**Published:** 2023-08-24

**Authors:** Janghyun Choi, Hansol Lee

**Affiliations:** Department of Biological Sciences, College of Natural Science, Inha University, Incheon, South Korea

**Keywords:** mesenchymal stem cells, osteogenic differentiation, NFIB, MLL1, Dlx5, stem cell maintenance, bivalent chromatin domain

## Abstract

Despite significant progress in our understanding of the molecular mechanism of mesenchymal stem cell (MSC) differentiation, less is known about the factors maintaining the stemness and plasticity of MSCs. Here, we show that the NFIB–MLL1 complex plays key roles in osteogenic differentiation and stemness of C3H10T1/2 MSCs. We find that depletion of either NFIB or MLL1 results in a severely hampered osteogenic potential and failed activation of key osteogenic transcription factors, such as Dlx5, Runx2, and Osx, following osteogenic stimuli. In addition, the NFIB–MLL1 complex binds directly to the promoter of *Dlx5*, and exogenous expression of Myc-Dlx5, but not the activation of either the BMP- or the Wnt-signaling pathway, is sufficient to restore the osteogenic potential of cells depleted of NFIB or MLL1. Moreover, chromatin immunoprecipitation (ChIP) and ChIP-sequencing analysis showed that the NFIB–MLL1 complex mediates the deposition of trimethylated histone H3K4 at both *Dlx5* and *Cebpa*, key regulator genes that function at the early stages of osteogenic and adipogenic differentiation, respectively, in uncommitted C3H10T1/2 MSCs. Surprisingly, the depletion of either NFIB or MLL1 leads to decreased trimethylated histone H3K4 and results in elevated trimethylated histone H3K9 at those developmental genes. Furthermore, gene expression profiling and ChIP-sequencing analysis revealed lineage-specific changes in chromatin landscape and gene expression in response to osteogenic stimuli. Taken together, these data provide evidence for the hitherto unknown role of the NFIB–MLL1 complex in the maintenance and lineage-specific differentiation of C3H10T1/2 MSCs and support the epigenetic regulatory mechanism underlying the stemness and plasticity of MSCs.

Mesenchymal stem cells (MSCs) are multipotent progenitor cells capable of self-renewal and differentiation into multiple lineages, such as osteogenesis and adipogenesis (1, 2, 3). The stemness, relatively easy isolation, and low immunogenicity have made MSCs a promising candidate for regenerative therapies (4, 5). Numerous studies have identified environmental and intracellular factors controlling the differentiation of MSCs that include lineage-specific transcription factors and epigenetic regulators (3, 6, 7). On the other hand, understanding of the factors maintaining the stemness and plasticity of MSCs is relatively limited compared to those identified in embryonic stem cells and induced pluripotent stem cells (8, 9, 10).

Osteogenic differentiation is initiated and maintained by the coordinated actions of lineage-specific transcription factors working at different stages of differentiation (11, 12). Among those are Runx2 and Osx, which play essential roles in forming the bone matrix and maintaining the osteoblastic phenotype by activating the expression of osteogenic-specific genes including *Ocn*, *Opn*, *Alp*, and *Col1a1* (13, 14). On the other hand, a homeobox-containing transcription factor Dlx5 contributes to the commitment and initiation of osteogenic differentiation by counteracting the inhibitory function of Msx2 and facilitating Runx2 expression (15, 16, 17, 18). In addition, studies have shown that the exogenous expression of Dlx5 promotes bone mineralization, whereas the loss of Dlx5 function results in decreased expression of genes involved in osteogenic differentiation and abnormality in bone formation (19, 20, 21, 22).

Lysine methylation of histones affects the chromatin function and is related to distinct biological events depending on the site and status of modification (23). Trimethylated histone H3K4 (H3K4me3) marks the transcription start site (TSS) of active genes. In contrast, the predominant occupancy of trimethylated histone H3K9 (H3K9me3) or trimethylated histone H3K27 at the promoter region is correlated with transcriptional silencing (24, 25). Histone lysine methyltransferases (KMTs) and histone demethylases (KDMs) determine the methylation status and have been implicated in osteogenic differentiation (26, 27). KMTs associated with repressive histone marks, such as H3K9-specific Setdb1 and G9a as well as H3K27-specific Ezh2, inhibit osteogenic differentiation, but their counteracting KDMs, including UTX and KDM4B, promote osteogenesis (28, 29, 30, 31, 32). In contrast, enzymes involved in the deposition and displacement of active histone marks have opposite effects on osteogenic differentiation. PHF20, an H3K4-specific KMT, promotes osteogenesis, but H3K4-specific KDMs, such as LSD1, KDM5A, and KDM5B, inhibit the osteogenic differentiation of MSCs (33, 34, 35).

MLL1, also known as KMT2A and a human homolog of yeast COMPASS/Set1, is a member of the mixed-lineage leukemia family of KMTs and mediates the deposition of H3K4me3 at the promoter of developmental regulators such as *Hox* genes (36, 37). The functions of MLL1 during embryonic and postnatal development have been highlighted in studies using KO mice, in which MLL1 is required for the definitive hematopoiesis and the maintenance of somatic stem cells such as adult hematopoietic stem cells, intestinal stem cells, postnatal neural stem cells (NSCs), and muscle satellite cells (38, 39, 40, 41, 42, 43). In addition, MLL1 has been implicated in the regulation of lineage-specific differentiation because it regulates the adipogenic differentiation of C3H10T1/2 MSCs and myogenic differentiation of C2C12 myoblast cells (43, 44). Although the molecular mechanisms underlying gene-specific transcriptional regulation by MLL1 are not fully understood, it is believed that MLL1 exerts its functions through interactions with sequence-specific DNA-binding transcription factors, such as Pax7 and nuclear factor I B (NFIB) (40, 44).

The NFIB is a member of the NFI family of proteins (NFIA, NFIB, NFIC, and NFIX) and functions as a DNA-binding transcription factor (45). Members of the NFI family are expressed ubiquitously but highly prominent in embryonic stem cells and progenitor cells within the central nervous system and skeletomuscular tissue (46). Studies have suggested that the NFIs play important roles during stem cell development and lineage-specific differentiation (47, 48). NFIA and NFIB regulate adipogenic differentiation by targeting key adipogenic genes such as *Pparg* and *Cebpa* in C2C12 myoblast cells, 3T3-L1 preadipocytes, and C3H10T1/2 MSCs (44, 49, 50). In contrast, NFIC inhibits adipogenic gene expression and instead targets *Osx* to regulate osteogenic differentiation (51, 52). In addition, NFIX has been implicated in myogenesis during development and regeneration (53, 54). Despite their important roles in lineage-specific differentiation, the understanding of the molecular mechanisms through which NFIs regulate lineage-specific transcription programs is limited. On the other hand, NFIB has recently been reported to form a functional complex with epigenetic factors such as MLL1 KMT and KDM4D KDM to regulate the adipogenic differentiation of C3H10T1/2 MSCs (44).

This study investigated the roles of NFIB and its interacting MLL1 in the osteogenic differentiation of C3H10T1/2 MSCs. The depletion of NFIB or knockdown of MLL1 resulted in severely inhibited differentiation, which could be rescued by the exogenous expression of Myc-Dlx5 but not by activating the Wnt- or BMP-signaling pathways. Furthermore, the NFIB–MLL1 complex targeted critical developmental genes, such as *Dlx5* and *Cebpa,* and helped establish the chromatin landscape consisting of H3K4me3/H3K9me3 in uncommitted C3H10T1/2 MSCs, which is likely to maintain MSCs in a ‘paused for responsive’ state until the relevant environmental signal is reached. Overall, these findings support the idea of epigenetic control in MSC maintenance and plasticity.

## Results

### NFIB–MLL1 complex is required for the osteogenic differentiation of C3H10T1/2 MSCs

In our previous study, we reported that NFIB interacts with the MLL1 KMT complex and KDM4D KDM to target the key adipogenic transcription factors genes, such as *Cebpa* and *Pparg*, and regulates adipogenic differentiation of C3H10T1/2 MSCs (44). The NFIB–MLL1 complex is required for the deposition of H3K4me3 at the promoter of *Cebpa* even in undifferentiated cells, and the depletion of either NFIB (shNFIB) or MLL1 (shMLL1) resulted in the decreased expression of genes involved in adipogenic differentiation. Of particular interest, RNA-seq analysis identified *Dlx5*, a well-known osteogenic transcription factor gene, as one of those affected by the depletion of NFIB or MLL1 in C3H10T1/2 MSCs prior to adipogenic stimuli (44). Given that C3H10T1/2 MSCs have multilineage differentiation potential and depletion of NFIB or MLL1 led to decreased expression of both adipogenic regulator *Cebpa* and osteogenic factor *Dlx5* in uncommitted MSCs, we investigated whether NFIB and its interacting partners, MLL1 and KDM4D, are involved in the regulation of osteogenic differentiation of C3H10T1/2 MSCs.

**Figure 1:**
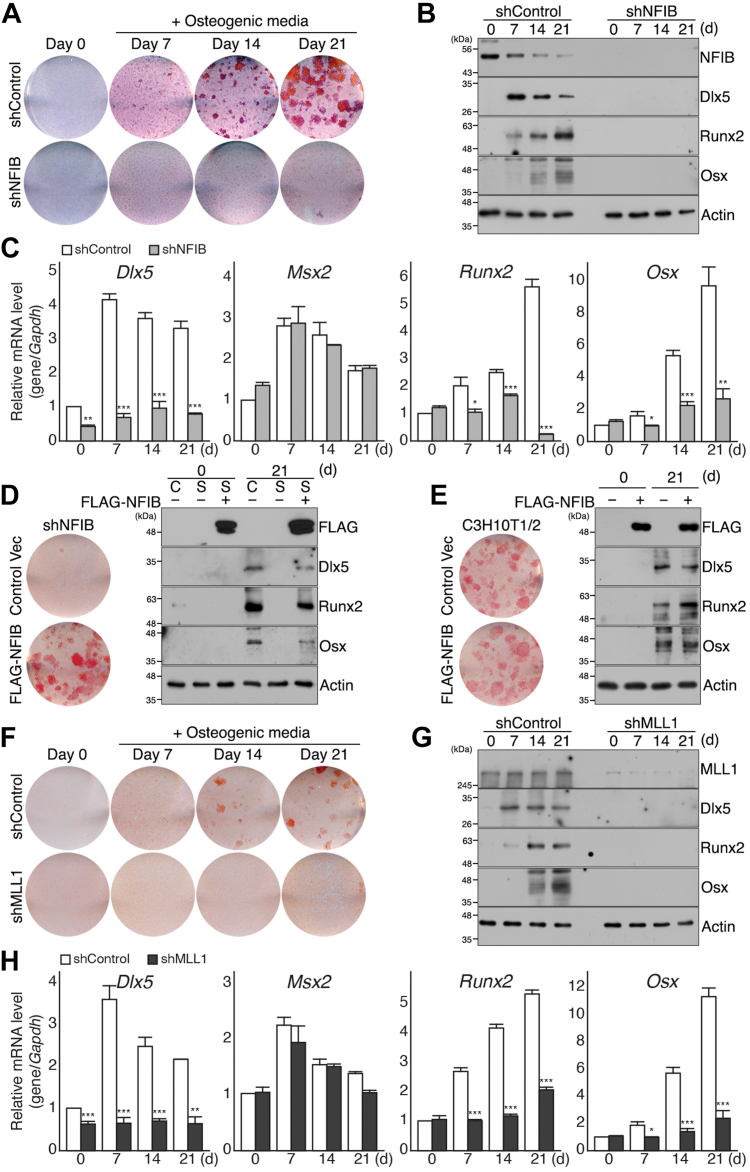
**NFIB and MLL1 are required for the osteogenic differentiation of C3H10T1/2 mesenchymal stem cells.***A*–*C*, C3H10T1/2 cells expressing control shRNA (shControl) or *Nfib* shRNA (shNFIB) were grown to confluence (day 0) and cultured in osteogenic medium containing 20 mM β-glycerophosphate, 100 μg/ml ascorbic acid 2-phosphate, and 0.1 μM dexamethasone up to 21 days. *A*, shControl and shNFIB cells were seeded into 6-well plates and cultured in an osteogenic medium. At indicated time points (0, 7, 14, and 21 days), cells were fixed and stained with Alizarin red S (ARS). Shown are representative data from at least five independent experiments. All images were obtained from a single round of experiments. *B*, immunoblot results of NFIB, Dlx5, Runx2, Osx, and Actin in shControl (*left*) or shNFIB cells (*right*). Actin was used as a loading control. Whole cell extracts (WCEs) were prepared at indicated time points (0, 7, 14, and 21 days). All images were from a single round of experiments. *C*, RT-qPCR analysis of *Dlx5*, *Msx2*, *Runx2*, and *Osx* expression in shControl (*white*) and shNFIB (*gray*) cells following osteogenic induction. For each gene, relative mRNA values were calculated as relative to shControl cells (day 0) after normalization to *Gapdh* mRNA. Data were analyzed using two-tailed Student’s *t* test and presented as mean ± SD (n = 3 independent experiments); ∗*p* < 0.05, ∗∗*p* < 0.01, ∗∗∗*p* < 0.001 *versus* control cells in each day. *D*, shControl (C) and shNFIB (S) cells were infected with retroviruses expressing FLAG-NFIB or empty vector (control vec) and cultured in an osteogenic medium for 21 days. Shown on the *left* are ARS staining results of shNFIB cells infected with the control vector (*upper*) and retrovirus expressing FLAG-NFIB (*lower*) after 21 days. Shown on the *right* are immunoblot results of FLAG epitope, Dlx5, Runx2, Osx, and Actin in shControl, shNFIB, and shNFIB cells expressing exogenous FLAG-NFIB. WCEs were prepared before (0) and after (21) osteogenic differentiation. Actin was used as a loading control. Images shown were obtained from a single round of experiments and a representative of at least three independent experiments. *E*, overexpression of FLAG-NFIB in control C3H10T1/2 MSCs. Shown on the *left* are ARS staining results of C3H10T1/2 MSCs infected with the control vector (*upper*) or retrovirus expressing FLAG-NFIB (*lower*) after 21 days. Shown on the *right* are immunoblot results of the FLAG epitope, Dlx5, Runx2, Osx, and Actin in control cells and cells expressing FLAG-NFIB. WCEs were prepared at day 0 and day 21 following osteogenic induction. Actin was used as a loading control. All images were obtained from a single round of experiments and a representative of at least three independent experiments. *F–H*, C3H10T1/2 MSCs expressing control shRNA (shControl) or *Mll1* shRNA (shMLL1) were cultured until confluence (day 0), and cells were then induced into osteogenic differentiation for 21 days. *F*, ARS staining results of shControl (*upper*) and shMLL1 cells (*lower*). Cells were fixed and stained before (day 0) and after (day 7, 14, and 21) osteogenic induction. Shown are the representative data of at least five independent experiments and all images were from a single round of experiments. *G*, immunoblot results of MLL1, Dlx5, Runx2, Osx, and Actin (loading control) in shControl (*left*) or shMLL1 cells (*right*) at indicated time points (0, 7, 14, and 21 days). The images were obtained from a single round of experiments and a representative of at least three independent experiments. *H*, RT-qPCR analysis of *Dlx5*, *Msx2*, *Runx2*, and *Osx* in shControl (*white*) and shMLL1 (*dark gray*) cells. For each gene, relative mRNA values were calculated as relative to shControl cells (day 0) following normalization to *Gapdh*. Data were analyzed using a two-tailed Student’s *t* test and presented as the mean ± SD (n = 3 independent experiments); ∗*p* < 0.05, ∗∗*p* < 0.01, ∗∗∗*p* < 0.001 *versus* control cells in each day. MSC, mesenchymal stem cell; RT-qPCR, quantitative reverse transcription-PCR.

We first examined the expression of the NFIB–MLL1 complex during osteogenic differentiation (Fig. S1, *A* and *B*). Unlike the increased expression during adipogenic differentiation of C3H10T1/2 MSCs (44), the expression of both NFIB and KDM4D decreased gradually following osteogenic stimuli in C3H10T1/2 MSCs (Fig. S1, *A* and *B*). On the other hand, no meaningful change in MLL1 expression was observed during the same period (Fig. S1, *A* and *B*). Next, we investigated whether the NFIB–MLL1 complex is required for the osteogenic differentiation of C3H10T1/2 MSCs. The depletion of endogenous NFIB severely hampered the osteogenic potential of MSCs (Fig. 1*A*), which is evident by the lack of calcium deposits even after 21 days of incubation in an osteogenic medium. Immunoblot and quantitative reverse transcription-PCR (RT-qPCR) analyses confirmed that the knockdown of NFIB led to inhibited or at least delayed expression of key pro-osteogenic regulators, such as Dlx5, Runx2, and Osx, but it had no significant effect on the level of anti-osteogenic regulator Msx2 (Fig. 1, *B* and *C*). In addition, the exogenous expression of FLAG-NFIB that lacks 3′ UTR targeted by shRNA restored the osteogenic potential in shNFIB cells, but the overexpression of FLAG-NFIB in the control C3H10T1/2 MSCs (shControl) failed to promote osteogenic differentiation (Fig. 1, *D* and *E*). Similarly, the knockdown of MLL1 (shMLL1) resulted in the loss of osteogenic potential in C3H10T1/2 MSCs and the inhibited or at least delayed induction of key osteogenic regulators (Fig. 1, *F*–*H*).

In addition to impaired osteogenic and adipogenic differentiation potentials, the depletion of NFIB or MLL1 appeared to affect the myogenic potential of MSCs because activation of myogenic regulatory factors, such as *Myod1* and *Myogenin*, was not observed in either shNFIB or shMLL1 cells following myogenic stimuli (Fig. S2). On the other hand, the depletion of KDM4D, which resulted in inhibited adipogenic differentiation, had no significant effects on the osteogenic differentiation of MSCs and led to the increased expression of myogenic regulatory factors following myogenic stimuli (Figs. S2 and S3). Of particular interest, despite the significant impacts on the lineage-specific differentiation potential, the depletion of NFIB or MLL1 had little effect on the proliferation of C3H10T1/2 MSCs (Fig. S4). Overall, these data demonstrate that the NFIB–MLL1 complex is necessary for C3H10T1/2 MSCs to maintain the multilineage differentiation potential, whereas KDM4D KDM might function differently in lineage-specific differentiation pathways.

### NFIB–MLL1 complex targets Dlx5 to regulate osteogenic differentiation in C3H10T1/2 MSCs

**Figure 2:**
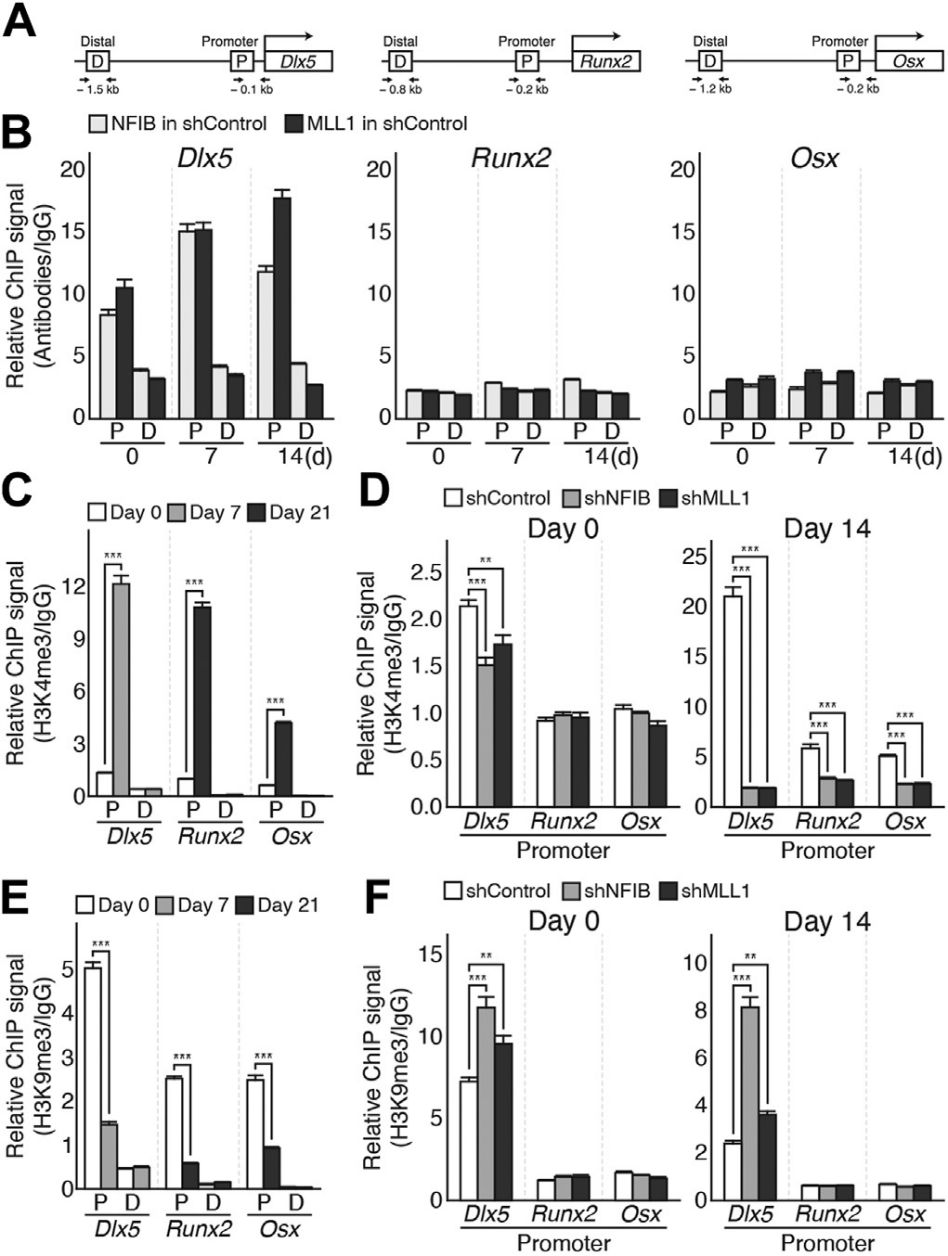
**NFIB/MLL1 complex targets Dlx5 to regulate the osteogenic differentiation of C3H10T1/2 MSCs.***A*, schematic representation of regions of *Dlx5*, *Runx2*, and *Osx* genes analyzed by ChIP-qPCR. P: promoter, D: distal region *B*, ChIP-qPCR analysis of NFIB (*light gray*) and MLL1 (*dark gray*) occupancies at the promoter (P) and distal region (D) of *Dlx5*, *Runx2*, and *Osx* genes in control C3H10T1/2 (shControl) cells. Chromatins were prepared from cells before (day 0)/after osteogenic induction (day 7 and 14) and then incubated with anti-NFIB, anti-MLL1, or IgG antibodies. Data were presented as mean ± SEM (n = 6 independent experiments). *C*, ChIP-qPCR analysis of trimethylated H3K4 (H3K4me3) enrichments at the promoter (P) and distal region (D) of *Dlx5*, *Runx2*, and *Osx* genes during osteogenic differentiation. Chromatins prepared at indicated time points (day 0, *white*; day 7, *gray*; day 21, *dark gray*) were subjected to immunoprecipitation with anti-H3K4me3 antibody or IgG. Data were analyzed using a two-tailed Student’s *t* test and presented as mean ± SEM (n = 3 independent experiments); ∗∗∗*p* < 0.001 *versus* day 0 of each locus. *D*, ChIP-qPCR analysis of H3K4me3 enrichments at the promoters of *Dlx5*, *Runx2*, and *Osx* in shControl (*white*), shNFIB (*gray*), and shMLL1 (*dark gray*) cells. Chromatins were prepared from cells before (day 0, *left*) and after osteogenic induction (day 14, *right*) and then immunoprecipitated with anti-H3K4me3 antibody or IgG. The data were analyzed using two-way ANOVA with Dunnett’s post hoc test (n = 6 independent experiments) and presented as mean ± SEM; ∗∗*p* < 0.01, ∗∗∗*p* < 0.001 *versus* shControl in each locus. *E*, ChIP-qPCR analysis of H3K9me3 changes at the *Dlx5*, *Runx2*, and *Osx* loci (P: promoter, D: distal region) during osteogenic differentiation of control C3H10T1/2 MSCs. Chromatins prepared at indicated time points (day 0, *white*; day 7, *gray*; day 21, *dark gray*) were immunoprecipitated with anti-H3K9me3 antibody or IgG. Data were analyzed using a two-tailed Student’s *t* test and presented as mean ± SEM (n = 3 independent experiments); ∗∗∗*p* < 0.001 *versus* day 0 of each locus. *F*, ChIP-qPCR analysis of H3K9me3 enrichments at the promoter and distal regions of *Dlx5*, *Runx2*, and *Osx* in shControl (*white*), shNFIB (*gray*), and shMLL1 (*dark gray*) cells during osteogenic differentiation. Immunoprecipitations were carried out using the chromatins prepared from cells before (day 0, *left*) and after osteogenic induction (day 14, *right*) with indicated anti-H3K9me3 or IgG antibodies. Data were analyzed using two-way ANOVA with Dunnett’s post hoc test (n = 6 independent experiments) and presented as mean ± SEM; ∗∗*p* < 0.01, ∗∗∗*p* < 0.001 *versus* shControl in each locus. For the relative ChIP signal described in (*B*–*F*), the percentage input (% input) was calculated for each sample and then presented as antibody signal (% input) divided by IgG signal (% input). ChIP, chromatin immunoprecipitation; H3K4me3, trimethylated histone H3K4; H3K9me3, trimethylated histone H3K9; MSC, mesenchymal stem cell.

We next investigated whether pro-osteogenic transcription factor genes, such as *Dlx5*, *Runx2*, and *Osx*, are targeted directly by the NFIB–MLL1 complex in C3H10T1/2 MSCs (Fig. 2*A*). Chromatin immunoprecipitation (ChIP) analysis showed that both NFIB and MLL1 were enriched at the *Dlx5* promoter but not at a distal region of the *Dlx5* gene in the control MSCs (Fig. 2*B*, *left*). Of particular interest, enrichments at the *Dlx5* promoter were observed not only in cells undergoing osteogenic differentiation but also in uncommitted cells (Fig. 2*B*, *left*). On the other hand, no meaningful binding of the NFIB–MLL1 complex was observed at genes involved in the later stages of osteogenic differentiation, such as *Runx2* and *Osx* (Fig. 2*B*, *middle* and *right*).

We next examined the occupancy of H3K4me3 at the promoter of osteogenic regulator genes because MLL1 is a core component of the MLL1 KMT complex that mediates H3K4me3 (Fig. 2, *C* and *D*). As expected, the H3K4me3 levels at the promoter of *Dlx5*, *Runx2*, and *Osx* were increased markedly in the control MSCs (shControl) following osteogenic stimuli (Fig. 2*C*). On the other hand, such increases were not observed in either shNFIB cells or shMLL1 cells (Fig. 2*D*, *right*). Interestingly, H3K4me3 levels at the *Dlx5* promoter were modestly but meaningfully lower in shNFIB and shMLL1 cells than those measured in shControl cells, which suggests that the NFIB–MLL1 complex may target the *Dlx5* promoter, even in uncommitted MSCs (Fig. 2*B*, *left*, and Fig. 2*D*, *left*).

The promoters of adipogenic regulator genes, such as *Cebpa* and *Pparg*, are marked by both active H3K4me3 and repressive H3K9me3 in C3H10T1/2 MSCs, and the depletion of NFIB or MLL1 disrupts the balance between those two histone modifications (44, 45). Hence, we tested whether the depletion of the NFIB–MLL1 complex affects the level of H3K9me3 at osteogenic regulator genes. As shown in Figure 2*E*, the level of H3K9me3 at the promoter of osteogenic regulator genes decreased following osteogenic stimuli, which appeared to be inversely correlated with the level of H3K4me3. Surprisingly, the levels of H3K9me3 at the *Dlx5* promoter in shNFIB and shMLL1 cells were significantly higher than in shControl cells, and these elevated H3K9me3 were observed even before osteogenic stimuli (Fig. 2*F*, *left*). In contrast, no significant changes were detected at the promoters of *Runx2* and *Osx* in shNFIB and shMLL1 cells (Fig. 2*F*). These data suggest that the NFIB–MLL1 complex directly targets the *Dlx5* promoter and deposits active chromatin marks, H3K4me3, which might be critical for maintaining the osteogenic potential of C3H10T1/2 MSCs.

### Exogenous expression of Dlx5 restores osteogenic potential in cells depleted of either NFIB or MLL1

**Figure 3:**
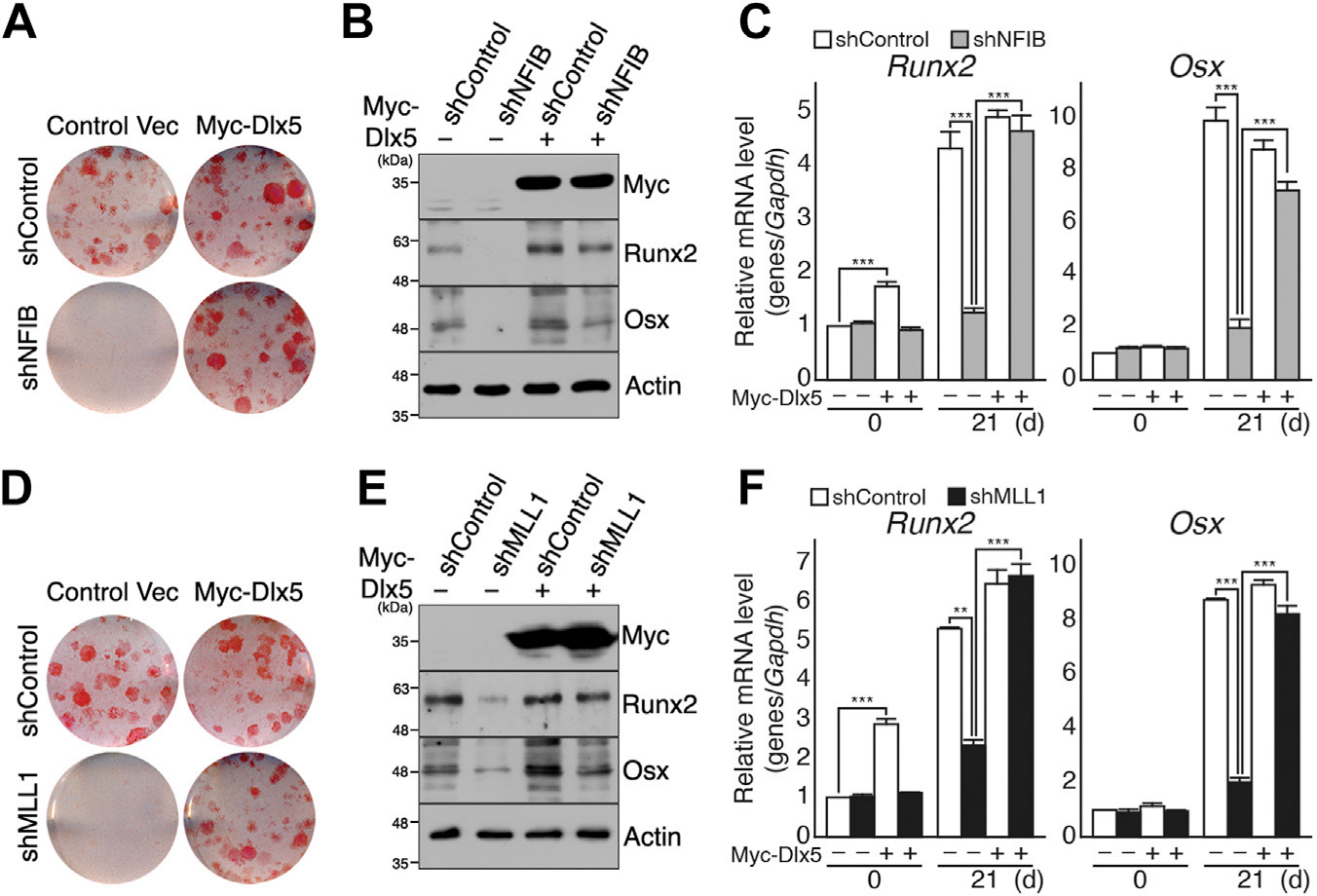
**Exogenous expression of Myc-Dlx5 rescues osteogenic defects in shNFIB and shMLL1 cells.***A–C*, shControl and shNFIB cells were infected with retroviruses expressing Myc-Dlx5 or empty vector (Control Vec) and then incubated in osteogenic medium containing 20 mM β-glycerophosphate, 100 μg/ml ascorbic acid 2-phosphate, and 0.1 μM dexamethasone for 21 days. *A*, ARS staining results of shControl (*upper*) and shNFIB cells (*lower*), infected with empty vector (*left*) or retrovirus expressing Myc-Dlx5 (*right*). Shown are representative data of at least five independent experiments and all images were from a single round of experiments. *B*, immunoblot results of Myc epitope, Runx2, Osx, and Actin in shControl and shNFIB cells, both of which were infected with empty vector (−) or retrovirus expressing Myc-Dlx5 (+). Actin was used as a loading control. The images shown were obtained from a single experiment and a representative of at least three independent experiments. *C*, RT-qPCR analysis of *Runx2* and *Osx* mRNAs to assess osteogenic differentiation in shControl (*white*) and shNFIB (*gray*) cells with or without exogenous Myc-Dlx5. The relative mRNA values for each gene were calculated as relative to the shControl cells (Day 0) after normalization to *Gapdh*. The data were analyzed using two-way ANOVA with Dunnett’s post hoc test (n = 3 independent experiments) and presented with mean ± SD. ∗∗∗*p* < 0.001. *D–F*, shControl and shMLL1 cells were infected with retroviruses expressing Myc-Dlx5 or empty vector. Infected cells were grown to confluence (day 0), and then osteogenic differentiation was induced by incubating in the presence of 20 mM β-glycerophosphate, 100 μg/ml ascorbic acid 2-phosphate, and 0.1 μM dexamethasone for 21 days (day 21). Osteogenic differentiation was assessed by ARS staining (*D*), immunoblotting (*E*), and RT-qPCR analysis (*F*). *D*, ARS staining results of shControl (*upper*) and shMLL1 cells (*lower*), infected with empty vector (*left*) or retrovirus expressing Myc-Dlx5 (*right*). The representative data of at least five independent experiments are shown, and all images were from a single round of experiments. *E*, immunoblot results of Myc epitope, Runx2, Osx, and Actin in shControl and shMLL1 cells, both of which were infected with an empty vector (−) or retrovirus expressing Myc-Dlx5 (+). Actin was used as a loading control. Images shown were obtained from a single round of experiments and a representative of at least three independent experiments. *F*, RT-qPCR analysis of *Runx2* and *Osx* mRNAs in shControl (*white*) and shMLL1 (*dark gray*) cells with or without exogenous Myc-Dlx5. For each gene, relative mRNA levels were calculated as relative to the shControl cells (day 0) after normalization to *Gapdh*. The data were analyzed using two-way ANOVA with Dunnett’s post hoc test (n = 3 independent experiments) and presented with mean ± SD. ∗∗*p* < 0.05, ∗∗∗*p* < 0.001. ARS, Alizarin Red S; RT-qPCR, quantitative reverse transcription-PCR.

We next tested whether the exogenous expression of Dlx5 would be sufficient to restore osteogenic potential in shNFIB and shMLL1 cells to confirm that defective osteogenic differentiation is directly related to the failed activation of Dlx5 upon osteogenic stimuli (Fig. 3). Indeed, strong Alizarin Red S (ARS) staining observed in both shNFIB and shMLL1 cells suggested that they could respond to osteogenic stimuli when infected with retrovirus expressing Myc-Dlx5 (Fig. 3, *A* and *D*). Immunoblot and RT-qPCR analyses confirmed that replenishing Dlx5 was sufficient for shNFIB (Fig. 3, *B* and *C*) and shMLL1 cells to regain osteogenic potential (Fig. 3, *E* and *F*). Of note, the exogenous expression of Myc-Dlx5 appeared to regulate Runx2 expression positively in undifferentiated MSCs, which aligns with previous reports (15, 18) (Fig. 3, *C* and *F*, *left*). These results demonstrate that the function of the NFIB–MLL1 complex in osteogenic differentiation is, at least in part, associated with its ability to control Dlx5 expression.

### Pro-osteogenic signals failed to rescue osteogenic defects in cells depleted of NFIB or MLL1

**Figure 4:**
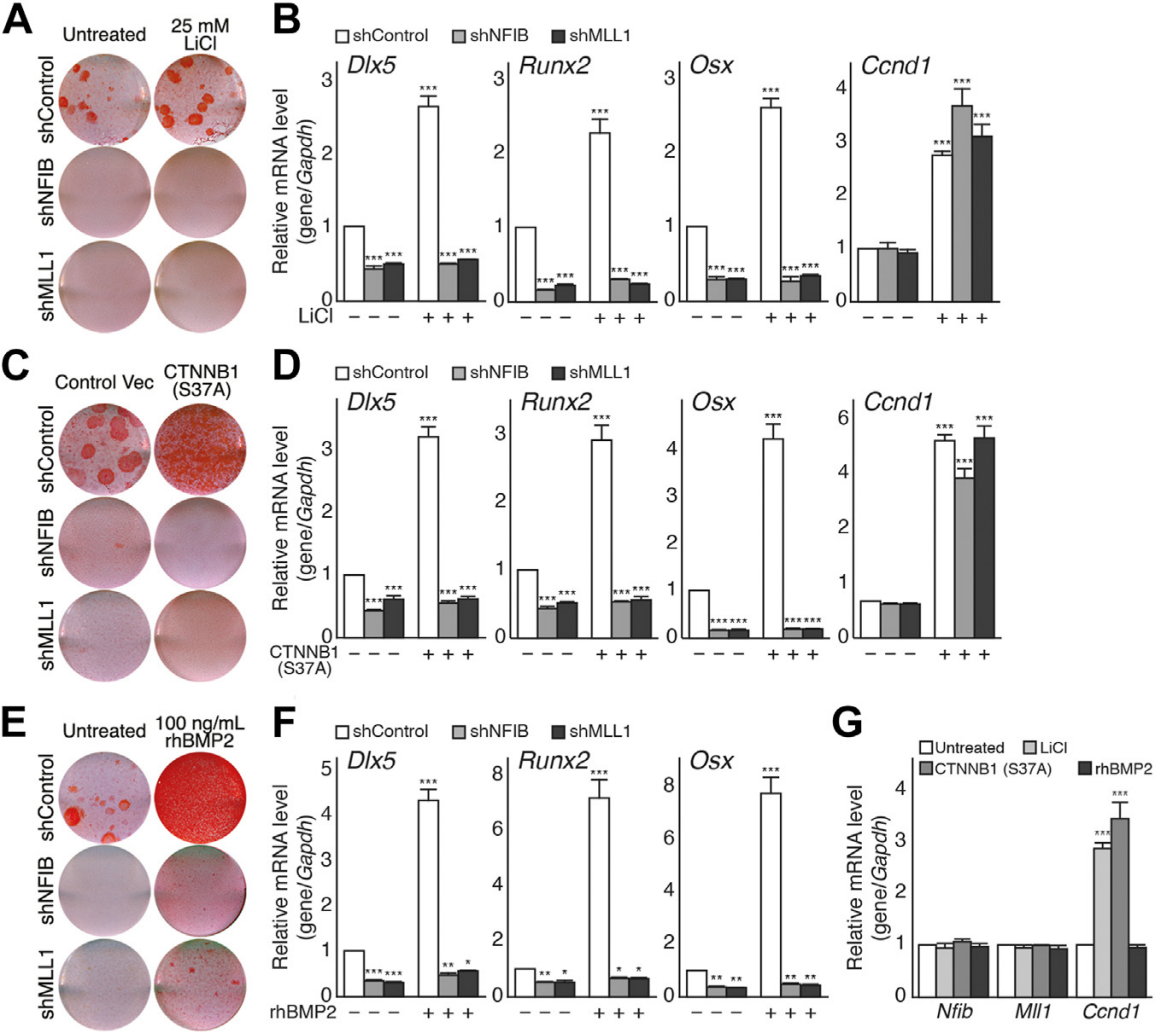
**Wnt- or BMP-signaling activation fails to overcome the osteogenic defects in shNFIB and shMLL1 cells.***A* and *B*, shControl, shNFIB, and shMLL1 cells were treated with 25 mM of LiCl for 24 h and further incubated in an osteogenic medium containing 20 mM β-glycerophosphate, 100 μg/ml ascorbic acid 2-phosphate, and 0.1 μM dexamethasone for 14 days. *A*, ARS staining results of untreated cells (*left*) and LiCl-treated cells (*right*) after 14 days of incubation in an osteogenic medium; shControl (*top*), shNFIB (*middle*), and shMLL1 (*bottom*). *B*, RT-qPCR results of *Dlx5*, *Runx2*, *Osx,* and *Ccnd1* in shControl (*white*), shNFIB (*gray*), and shMLL1 (*dark gray*) cells. The mRNA level of cyclin D1 (*Ccnd1*) was measured to confirm the activation of the Wnt-signaling pathway. For each gene, relative mRNA levels were calculated as relative to untreated (−) shControl cells after normalization to *Gapdh*. *C* and *D*, shControl, shNFIB, and shMLL1 cells were infected with retrovirus expressing a constitutively active mutant of β-catenin (CTNNB1, S37A) or control vector and then cultured in an osteogenic medium for 14 days. *C*, ARS staining results of shControl (*top*), shNFIB (*middle*), and shMLL1 (*bottom*) cells infected with empty retroviral vector (*left*) or retrovirus expressing a mutant CTNNB1 (S37A) (*right*). *D*, RT-qPCR results of *Dlx5*, *Runx2*, *Osx,* and *Ccnd1* in shControl (*white*), shNFIB (*gray*), and shMLL1 (*dark gray*) cells. The mRNA levels of cyclin D1 (*Ccnd1*) were measured to monitor Wnt-signaling activation. For each gene, relative mRNA levels were calculated as relative to the shControl cells infected with empty vector (−) following normalization to *Gapdh*. *E* and *F*, shControl, shNFIB, and shMLL1 cells were cultured in osteogenic media with or without 100 ng/ml of recombinant human BMP2 (rhBMP2) for 14 days. *E*, ARS staining results of untreated (*left*) or rhBMP2-treated (*right*) shControl (*top*), shNFIB (*middle*), and shMLL1 (*bottom*) cells after 14 days of osteogenic induction. *F*, RT-qPCR results of *Dlx5*, *Runx2*, and *Osx* in shControl (*white*), shNFIB (*gray*), and shMLL1 (*dark gray*) cells. For each gene, relative mRNA levels were calculated as relative to untreated shControl cells (−) following normalization to *Gapdh*. For (*B*, *D*, and *F*), data were analyzed using two-way ANOVA with Dunnett’s post hoc test (n = 3 independent experiments) and presented with mean ± SD. ∗*p* < 0.05, ∗∗*p* < 0.01, ∗∗∗*p* < 0.001 *versus* untreated/empty vector–infected shControl cells (−). *G*, C3H10T1/2 cells were treated with 25 mM of LiCl (*light gray*), infected with retrovirus expressing constitutively active mutant of β-catenin (S37A), or cultured in the presence of 100 ng/ml rhBMP2 (*dark gray*). Shown are the RT-qPCR results of *Nfib*, *Mll1*, and *Ccnd1* after 14 days of incubation in an osteogenic medium. For each gene, data were analyzed using two-way ANOVA with Dunnett’s post hoc test (n = 3 independent experiments) and presented with mean ± SD. ∗∗∗*p* < 0.001 *versus* untreated shControl cells (*white*). ARS, Alizarin Red S; LiCl, lithium chloride; RT-qPCR, quantitative reverse transcription-PCR.

Dlx5 is one of the earliest working transcription factors that functions at the commitment and initiation stage of osteogenic differentiation for its immediate and robust expression following pro-osteogenic Wnt/BMP signals (55, 56). Because both pro-osteogenic signals and the NFIB–MLL1 complex regulate Dlx5 expression, we examined whether the activation of pro-osteogenic Wnt- or BMP-signaling pathway could override defects in osteogenic differentiation in shNFIB and shMLL1 cells (Fig. 4). As expected, the exogenous expression of a constitutively active β-catenin mutant (S37A) (Fig. 4, *A* and *B*) or treatment with 25 mM of lithium chloride (Fig. 4, *C* and *D*), which are well-established ways to activate the Wnt signaling pathway in cells, promoted osteogenic differentiation of control MSCs, as shown by the stronger ARS staining and higher expression of the key osteogenic regulators in cells with Wnt-activating treatment (Fig. 4, *A*–*D*). On the other hand, despite marked increases in *Ccnd1* expression following Wnt-activating treatments, no meaningful changes were observed in either shNFIB or shMLL1 cells (Fig. 4, *A* and *B*). Similarly, the control MSCs exhibited accelerated osteogenic differentiation in the presence of recombinant BMP2, but the same treatment failed to overcome the osteogenic defects in shNFIB and shMLL1 cells (Fig. 4, *E* and *F*). More importantly, unlike Dlx5 showing dramatic increases in expression by activation of the Wnt/BMP signaling pathways, these pro-osteogenic stimuli had no significant effects on the steady-state mRNA level of either *Nfib* or *Mll1*, suggesting that the NFIB–MLL1 complex may function independently from the Wnt/BMP signaling pathways in osteogenic differentiation of C3H10T1/2 MSCs (Fig. 4*G*). We also examined whether inhibition of the Wnt signaling pathway, which can be achieved by treatment with an NSC668063, a *Dvl* inhibitor, or the exogenous expression of FLAG-Axin1 rescues adipogenic defects in shNFIB and shMLL1 cells (Fig. S5). Inhibition of the Wnt signaling pathway led to increased amounts of lipid droplets (Fig. S5, *A* and *C*) as well as the expression of key adipogenic markers, such as *Cebpa*, *Pparg*, and *Fabp4*, suggesting positive regulation of adipogenic differentiation in C3H10T1/2 MSCs (Fig. S5, *B* and *D*). On the other hand, the same treatments resulted in no meaningful restoration of the adipogenic differentiation potential in shNFIB or shMLL1 cells (Fig. S5). These results suggest that the functions of the NFIB–MLL1 complex may be to maintain the stemness and plasticity of the MSCs rather than regulate differentiation into a specific lineage.

### NFIB–MLL1 complex contributes to the chromatin landscape in uncommitted C3H10T1/2 MSCs

**Figure 5:**
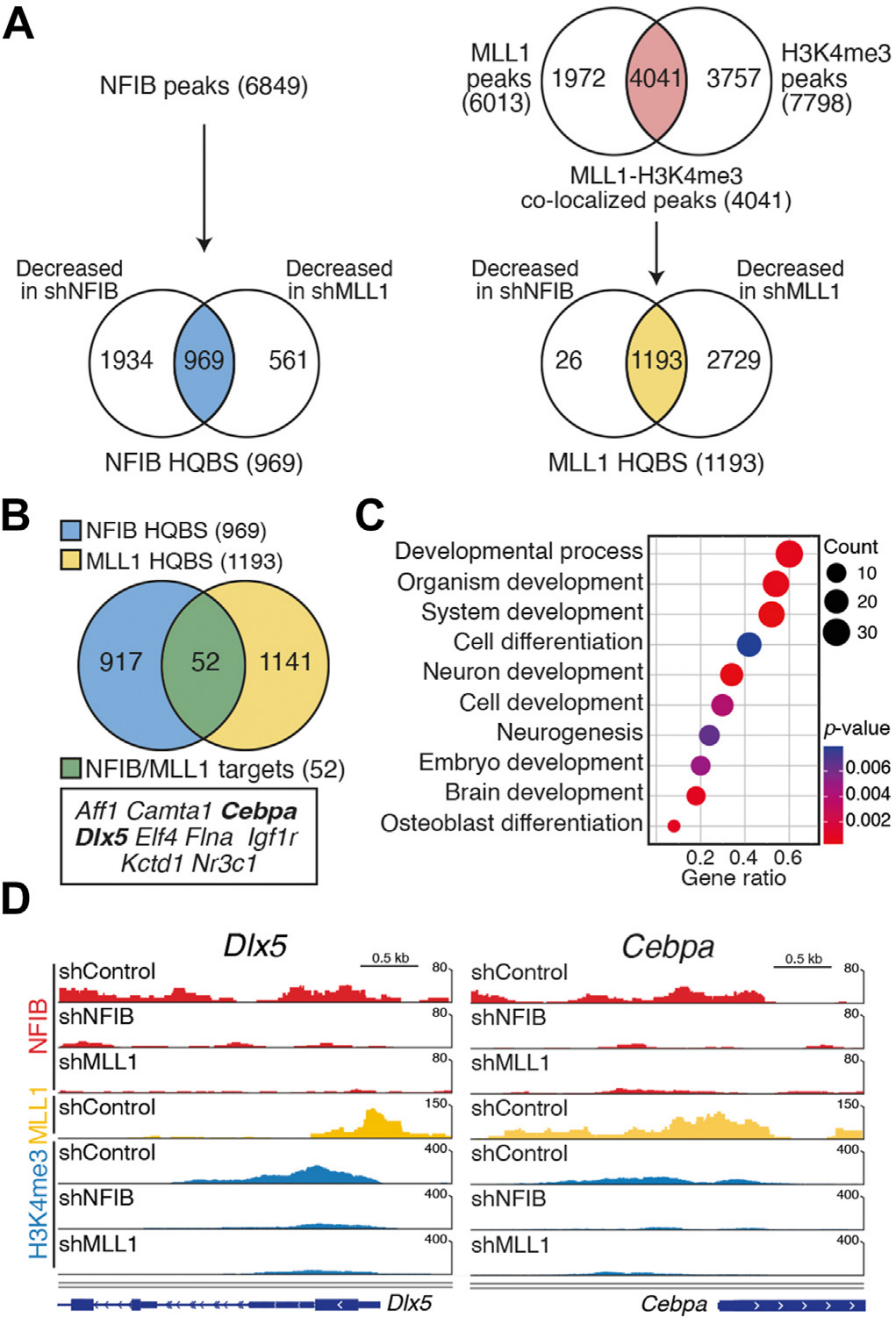
**NFIB/MLL1 complex targets developmental genes in uncommitted C3H10T1/2 MSCs.***A*, flowchart showing the processes for identifying NFIB high-quality binding sites (HQBS, *left*) and MLL1 HQBS (*right*) from ChIP-seq peak-call analysis. The Venn diagram on the *lower left* shows NFIB HQBS (n = 969, *blue*), which are the NFIB peaks affected by NFIB or MLL1 depletion in C3H10T1/2 MSCs. The Venn diagram on the *upper right* shows MLL1-H3K4me3 colocalized peaks (n = 4,041, *red*) that are included in both 6013 MLL1 peaks and 7798 H3K4me3 peaks identified in uncommitted MSCs. The Venn diagram on the *lower right* shows MLL1 HQBS (n = 1,193, *yellow*), which are MLL1-H3K4me3 colocalized peaks affected by NFIB or MLL1 knockdown. *B*, Venn diagram (*upper*) shows NFIB/MLL1 targets (n = 52, green), commonly included in 969 NFIB HQBS and 1193 MLL1 HQBS. The *lower box* shows a list of representative transcription factor genes including *Cebpa* and *Dlx5*. *C*, gene ontology (GO) classification showing the biological process (BP) terms of NFIB/MLL1 targets (n = 52). The size of bubble indicates the number of genes. Color depth indicates significance (*p* < 0.05). The x-axis represents enrichment abundance (hit genes/populated genes) and the y-axis shows different GO terms. *D*, Interactive Genomics Viewer (IGV) genome browser snapshots of *Dlx5* (*left*) and *Cebpa* (*right*) showing tracks of NFIB (*red*), MLL1 (*yellow*), and H3K4me3 (*blue*) ChIP-seq tag counts in shControl, shNFIB, and shMLL1 cells before osteogenic induction. ChIP, chromatin immunoprecipitation; ChIP-seq, ChIP-sequencing; H3K4me3, trimethylated histone H3K4; MSC, mesenchymal stem cell.

Previous results suggested that the functions of the NFIB–MLL1 complex are likely to be related to its DNA binding and histone modification activities (Fig. 2) (44). Therefore, we next conducted ChIP-sequencing (ChIP-seq) analysis using the control C3H10T1/2 MSCs and cells expressing specific shRNAs (shNFIB and shMLL1) to explore the direct targets of NFIB and examine the genome-wide colocalization of NFIB and MLL1 (Figs. 5 and S6). In the uncommitted control C3H10T1/2 MSCs, 32,934 NFIB binding peaks were identified. Among those 32,934 peaks, 9185 peaks were located at the promoter region (within ± 3 kb from TSSs) and 6849 NFIB peaks out of those 9185 peaks were enriched at the proximal promoter region (within ± 1 kb from TSSs) (Figs. 5*A* and S6*A*).

The genome-wide impact of NFIB or MLL1 depletion on NFIB occupancy in C3H10T1/2 MSCs was analyzed (Fig. 5*A*). As a result, 2903 and 1530 peaks of 6849 NFIB-binding peaks were affected by NFIB or MLL1 knockdown, respectively (Fig. 5*A*. *left* and Fig. S6*D*, *left*), and 969 peaks, which were designated as NFIB high-quality binding sites (HQBS), were commonly presented (Fig. 5*A*, *left*). Using similar approaches, 20,363 MLL1 binding peaks and 19,487 H3K4me3-enriched peaks were identified, of which 6013 and 7798 peaks were localized at proximal promoter region in uncommitted C3H10T1/2 MSCs, respectively (Fig. 5*A*, *right*, Fig. S6, *B* and *C*). More than 60% of the MLL1 binding peaks (4041/6013) were included in the list of H3K4me3-enriched peaks (Fig. 5*A*, *right*). Among the 4041 peaks, the depletion of NFIB or MLL1 led to a significant decrease in the H3K4me3 level at 1219 and 3922 peaks, respectively (Fig. 5*A*, *right* and Fig. S6*D*, *right*). Of note, 1193 peaks were commonly presented and designated as MLL1 HQBS (Fig. 5*A*, *right*). Finally, 52 NFIB/MLL1 direct targets listed in 969 NFIB HQBS and 1192 MLL1 HQBS were identified (Fig. 5*B*, *green* and Fig. S6*E*).

Gene ontology (GO) analysis of 52 NFIB/MLL1 direct targets (30/52) revealed a strong association with differentiation and development, such as developmental process (GO:0032502), cell differentiation (GO:0030154), neuron development (GO:0048666), and positive regulation of osteoblast differentiation (GO:0045669) (Fig. 5*C*). Surprisingly, both *Dlx5* and *Cebpa* were among those 52 NFIB/MLL1 targets (Fig. 5, *C* and *D*). Of note, GO analysis of 917 targets exclusive to NFIB HQBS (Fig. 5*B*, *blue*) identified nervous system development (GO:0007399), multicellular organism development (GO:0007275), and learning (GO:0007612) (Fig. S6*F*). On the other hand, the analysis of 1141 targets exclusive to MLL1 HQBS (Fig. 5*B*, *yellow*) failed to identify the GO terms related to differentiation and development (Fig. S6*G*). Overall, these data suggest that the functions of the NFIB–MLL1 complex in MSCs maintenance are associated with its selective targeting of developmental regulator genes such as *Dlx5* and *Cebpa* in uncommitted C3H10T1/2 MSCs.

### Osteogenic stimuli led to lineage-specific changes in NFIB binding and H3K4me3 deposition in C3H10T1/2 MSCs

**Figure 6:**
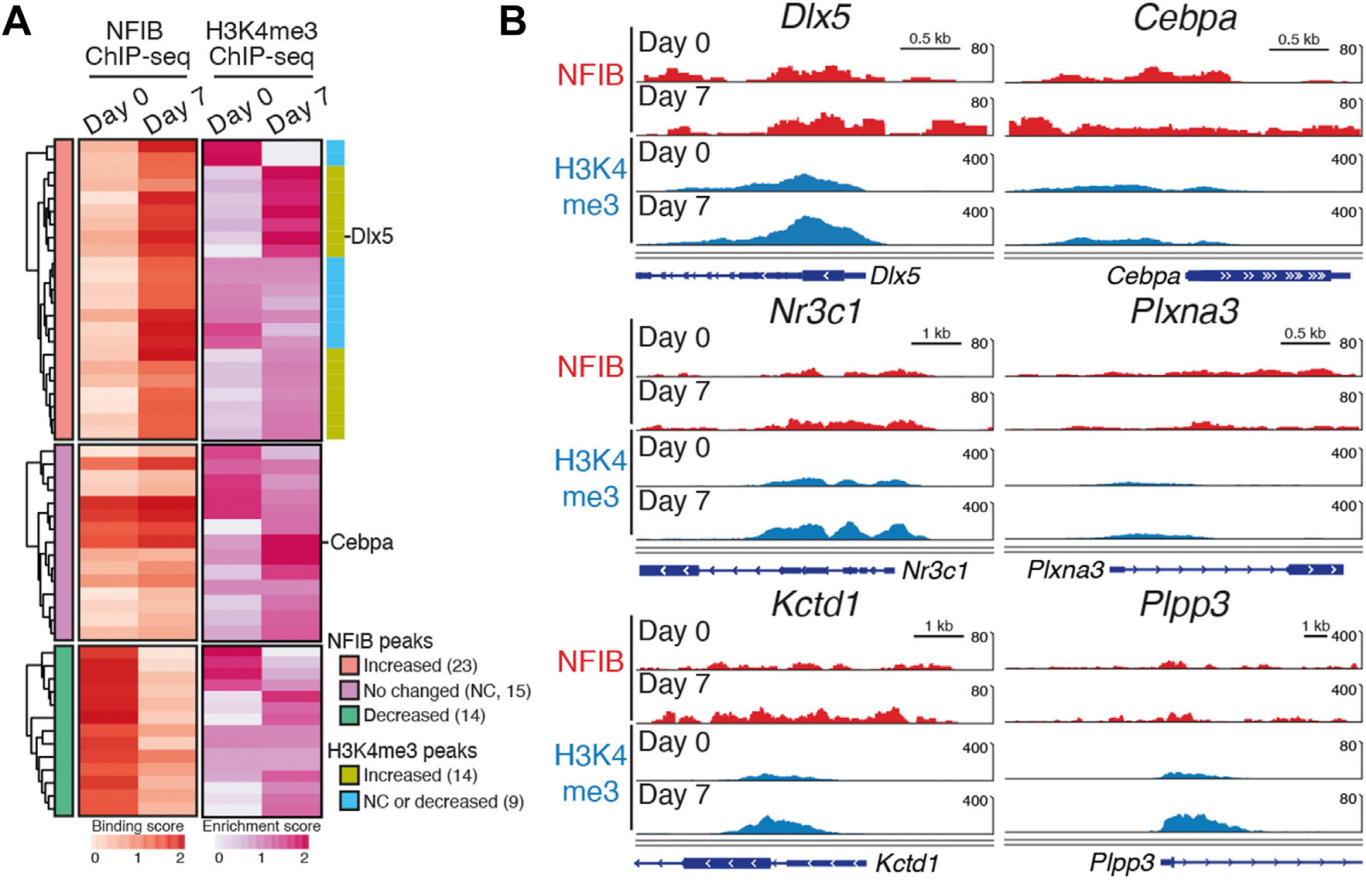
**Osteogenic stimuli lead to lineage-specific changes in NFIB binding and H3K4me3 deposition.***A*, heatmap showing 52 NFIB/MLL1 targets clustered into groups based on changes in NFIB ChIP-seq counts and H3K4me3 ChIP-seq counts in response to osteogenic stimuli (day 0 *versus* day 7). Shown on the *left* were 52 targets clustered into three groups based on NFIB ChIP-seq count changes following osteogenic stimuli: increased (n = 23, *red*), no change (n = 15, *purple*), and decreased (n = 14, *green*). A group exhibiting increased NFIB ChIP-seq counts (n = 23) was analyzed further based on changes in H3K4me3 ChIP-seq counts and presented with two different colors on the *right*: increase in H3K4me3 deposition (n = 14, *light green*) and decrease/no change (n = 9, *light blue*). The scale bar at the *bottom* of the heatmap represents the binding/enrichment score obtained using sicer2-df. *B*, IGV genome browser snapshots showing tracks of NFIB (*red*) and H3K4me3 (*blue*) peaks at representative genes demonstrating increased NFIB binding and H3K4me3 deposition in response to osteogenic stimuli (*Dlx5, Nr3c1, and Kctd1*) and genes showing no correlative increases (*Cebpa, Plxna3, and Plpp3*). ChIP, chromatin immunoprecipitation; ChIP-seq, ChIP-sequencing; H3K4me3, trimethylated histone H3K4.

Considering that osteogenic *Dlx5* and adipogenic *Cebpa* are targeted by the NFIB–MLL1 complex and enriched with H3K4me3 in uncommitted MSCs, we next investigated whether lineage-specific osteogenic stimuli affect the 52 NFIB/MLL1 direct targets differently on their NFIB occupancy and H3K4me3 deposition. Comparative ChIP-seq analysis of the 52 direct targets identified 14 genes that exhibited increased NFIB occupancy and elevated H3K4me3 deposition at the promoters following osteogenic stimuli (Fig. 6, *A* and *B*). More importantly, *Dlx5* was included in the list of 14 responsive genes, but adipogenic *Cebpa* was among those 38 nonresponsive genes (Fig. 6, *A* and *B*). These findings strongly suggest that the NFIB–MLL1 complex targets in uncommitted MSCs may respond differently to lineage-specific differentiation signals.

### NFIB–MLL1 complex regulates lineage-specific changes in transcriptome of C3H10T1/2 MSCs

**Figure 7:**
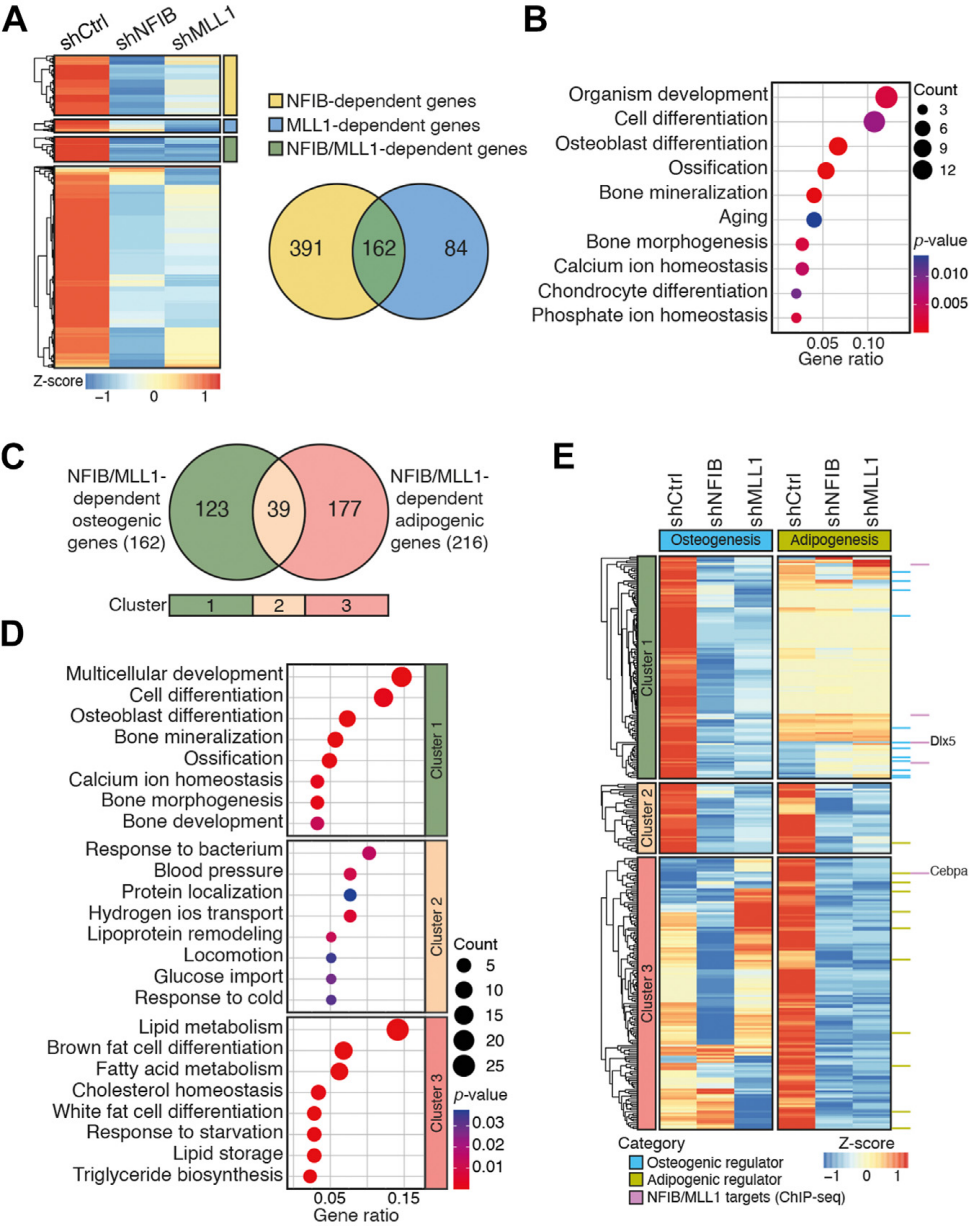
**NFIB/MLL1 complex regulates the lineage-specific transcriptome changes in C3H10T1/2 MSCs.***A*, shown on the *left* are hierarchical clustering results of 1962 osteogenesis-associated genes (OAGs) in shControl, shNFIB, and shMLL1 cells. One thousand nine hundred sixty-two OAGs were differentially expressed genes with |log2 fold change| >1 and FDR <0.05 upon osteogenic stimuli in shControl cells. OAGs are clustered into four groups and manually curated based on NFIB, MLL1 dependency, or both upon osteogenic stimuli: OAGs that failed to be activated only in shNFIB cells (NFIB-dependent genes, *yellow*), OAGs whose expressions were significantly decreased in shMLL1 cells but not in shNFIB cells (MLL1-dependent genes, *blue*), OAGs showing NFIB and MLL1 dependency (NFIB/MLL1-dependent genes, *green*), and OAGs with no significant dependency. The scale bar is based on the Z-score of log2 (CPM + 1). On the *right* is a Venn diagram depicting 162 NFIB/MLL1-dependent OAGs (*green*) commonly presented in 553 NFIB-dependent OAGs and 246 MLL1-dependent OAGs. *B*, gene ontology (GO) classification showing the biological process (BP) terms of 162 NFIB/MLL1-dependent OAGs with most genes annotated. The size of bubble indicates the number of genes. Color depth indicates significance (*p* < 0.05). The x-axis represents enrichment abundance (hit genes/populated genes) and the y-axis represent different GO terms. *C*, Venn diagram representing comparative analysis results of 162 NFIB/MLL1-dependent OAGs and 216 NFIB/MLL1-dependent adipogenesis-associated genes (AAGs, GSE131369), which were categorized into three groups based on lineage specificity: presented only in the list of 162 NFIB/MLL1-dependent OAGs (cluster 1, n = 123, *green*), presented only in NFIB/MLL1-dependent AAGs (cluster 3, n = 177, *red*), and genes found in both cluster 1 and cluster 3 (cluster 2, n = 39, *orange*). *D*, GO analysis classifications showing enriched functions of three clustered groups identified in (*C*). The size of bubble indicates the number of genes. Color depth indicates significance (*p* < 0.05). The x-axis represents enrichment abundance (hit genes/populated genes) and the y-axis shows different GO terms. *E*, heatmap showing the expression profile of 339 genes identified in (*C*) in shControl, shNFIB, and shMLL1 cells prior to/after induction of lineage-specific differentiation. The *light blue* and *light green bar* on the *right* next to the *box* indicate osteogenic and adipogenic regulators, respectively, and the *purple bars* on the rightmost depicts genes, including *Dlx5* and *Cebpa*, which are presented in the list of NFIB/MLL1 direct targets. The scale bar is based on the Z-score of log2 (CPM + 1). FDR, false discovery rate; MSC, mesenchymal stem cell.

We next conducted RNA-seq analysis of six different samples (shControl, shNFIB, and shMLL1 cells at day 0 and day 7) to investigate the roles of the NFIB–MLL1 complex on the transcriptome of C3H10T1/2 cells and identify the NFIB/MLL1-dependent genes during osteogenic differentiation (Figs. 7 and S7). By applying the criteria of greater than a two-fold change and a false discovery rate < 0.05, 3882 differentially expressed genes (DEGs; 1962 upregulated and 1920 downregulated) following osteogenic stimuli were identified in the control C3H10T1/2 MSCs and designated as osteogenesis-associated genes (OAGs) (Fig. S7*A*). GO analysis of those 1962 upregulated OAGs identified GO terms including cell differentiation (GO:0030154), osteoblast differentiation (GO:0001649), and bone mineralization (GO:0030282) (Fig. S7*B*, *left*). On the other hand, an analysis of 1920 downregulated OAGs revealed enrichment of the genes involved in the cell cycle process (Fig. S7*B*, *right*). In addition, cluster analysis of 1962 upregulated OAGs identified 553 NFIB-dependent genes and 246 MLL1-dependent genes (Fig. 7*A*). Further analysis showed that 65.8% (162/246) of MLL1-dependent OAGs were NFIB-dependent (Fig. 7*A*). Importantly, both *Dlx5* and *Runx2* were among those 162 NFIB/MLL1-dependent OAGs. (Fig. 7*A* and Data S1). GO analysis of 162 NFIB/MLL1-dependent OAGs revealed a strong association with osteoblast differentiation and bone mineralization (Fig. 7*B*). On the other hand, no GO terms associated with osteogenic differentiation were identified from analyses of 391 exclusively NFIB-dependent genes and 84 exclusively MLL1-dependent genes (Fig. 7*A*, *right* and Fig. S7, *C* and *D*). We also identified 305 NFIB-dependent, 142 MLL1-dependent, and 77 NFIB/MLL1-dependent genes from the analysis of 1920 downregulated OAGs (Fig. S7*E*). Unlike the 162 upregulated NFIB/MLL1-dependent genes, the analysis of 77 downregulated genes provided no meaningful information related to osteogenic differentiation (Fig. S7*F*).

Furtherly, comparative analysis of 162 NFIB/MLL1-dependent OAGs with 216 previously identified NFIB/MLL1-dependent adipogenesis-associated genes (AAGs) was conducted (44). We found that 123 out of 162 OAGs were exclusively presented in the list of OAGs, whereas 39 genes were commonly presented in both 162 OAGs and 216 AAGs (Fig. 7*C*). GO analyses showed a strong correlation with osteoblast differentiation of 123 OAGs (Fig. 7*D*, cluster 1) and an association with fat cell differentiation (Fig. 7*D*, cluster 3). On the other hand, no meaningful information related to either osteogenesis or adipogenesis was obtained from the analysis of 39 commonly presented OAGs/AAGs (Fig. 7*D*, cluster 2). Lastly, 52 NFIB–MLL1 complex targets from the ChIP-seq analysis were compared with 162 OAGs and 216 AAGs (Fig. 7*E*). Surprisingly, only five genes out of 52 direct targets were presented in the list of either 162 OAGs or 216 AAGs. Moreover, both *Dlx5* and *Cepba*, but no other known lineage-specific regulators, were included in those five genes (Fig. 7*E*). Collectively, these data show that the function of the NFIB–MLL1 complex in C3H10T1/2 MSCs is, at least in part, to serve as an intrinsic regulatory factor maintaining stemness and plasticity by contributing to chromatin landscape at the critical developmental genes such as *Dlx5* and *Cebpa*.

## Discussion

Despite the significant advances in understanding the roles of sequence-specific transcription factors and their interacting epigenetic modifiers in lineage-specific differentiation, relatively less is known about how they are integrated into the regulation of stemness and plasticity of MSCs. This study found that NFIB and its interacting MLL1 KMT control the expression of Dlx5 and thus regulate the osteogenic differentiation of C3H10T1/2 MSCs. Moreover, the current study supports the idea that roles of the NFIB–MLL1 complex may not be limited to a specific differentiation pathway but extended to maintaining the stemness and plasticity of MSCs, which involves targeting the developmental genes, such as *Dlx5* and *Cebpa*, and contribution to the establishment of bivalent chromatin domain consisting of H3K4me3/H3K9me3.

This study revealed the regulatory function of the NFIB–MLL1 complex in the osteogenic and likely the myogenic differentiation of C3H10T1/2 MSCs, which makes the NFIB–MLL1 complex a factor required for the trilineage differentiation potential of MSCs (Figs. 1 and S2) (44). The inhibited osteogenic differentiation in NFIB- or MLL1-knockdown cells and the restored osteogenic potential by exogenous expression of FLAG-NFIB in shNFIB cells supported roles of NFIB and MLL1 in osteogenic differentiation of MSCs (Fig. 1). Of note, exogenous expression of FLAG-NFIB failed to rescue osteogenic defects in cells depleted of MLL1 (data not shown), which is consistent with the previous report showing interdependency between NFIB and MLL1 in binding to genomic targets (44). Therefore, the functions of NFIB are likely to be mediated by interaction with MLL1 histone methyltransferase at least in C3H10T1/2 MSCs.

The selective interaction of NFIB with MLL1, but not with MLL3, has been reported previously (44). On the other hand, few studies have examined the isoform-dependent functions of NFIB, which has at least five different isoforms (isoforms 1–5). Because shRNA used for the generation of NFIB-depleted MSCs targets 3′ UTR of *Nfib gene* shared by all five isoforms and anti-NFIB antibodies used for ChIP and ChIP-seq analyses also recognize all five isoforms, results from the current study are not enough to determine the isoform dependency of NFIB in regulating the differentiation in C3H10T1/2 MSCs. However, it should be noted that FLAG-NFIB, which successfully rescued defects in lineage-specific differentiation, is derived from the *Nfib* isoform 3 (57). Although speculative, these data suggest that there might be an isoform-specific function of NFIB or at least functional redundancy among NFIB isoforms.

Although a plethora of osteogenic regulatory factors have been identified and described in the literature, most regulate the expression and function of Runx2 and Osx. Few studies have been conducted on regulating Dlx5 expression (12, 13, 20). This study identifies the NFIB–MLL1 complex as a specific regulator of Dlx5, for its selective binding to the *Dlx5* promoter and the sufficiency of exogenous Myc-Dlx5 in rescuing osteogenic defects in shNFIB and shMLL1 cells (Figs. 2 and 3). Of particular interest, RNA-seq analyses showed that *Dlx5* does not respond to adipogenic stimuli but only to osteogenic stimuli and thus is considered an exclusively osteogenic gene (Fig. 7). In addition, it has been reported that the *Dlx5* KO mice exhibit primarily bone-related defects but no significant adipogenesis/myogenesis-associated abnormalities (21, 22). Therefore, functions of Dlx5 are likely to be limited to osteogenic differentiation but not to adipogenic/myogenic differentiation in MSCs.

Biochemical analysis of the NFIB–MLL1 complex during osteogenic differentiation can be simply interpreted as the NFIB–MLL1 complex being integrated into the osteogenic transcription program working as an upstream regulator of Dlx5. However, evidence suggests that the complex instead functions as a maintenance factor for the stemness and plasticity of C3H10T1/2 MSCs rather than acts as a lineage-specific transcription regulator. First, unlike lineage-specific regulators that often exhibit reciprocal activity toward differentiation into other lineages (7, 58), depletion of the NFIB–MLL1 complex inhibits differentiation into osteogenic, adipogenic, and even possibly myogenic lineages, which is a common feature of MSCs defective of stemness and plasticity (9, 59, 60). Second, expression of Dlx5 increases robustly by pro-osteogenic BMP2- or Wnt-signaling activation (Fig. 4) and similarly, inhibition of Wnt-signaling pathway, which acts as a pro-adipogenic stimuli, markedly increases in the expression of adipogenic genes, such as *Cebpa* and *Pparg* (Fig. S5). However, modulation of extracellular signaling pathways had no effects on the expression of *Nfib* and *Mll1*. Moreover, they failed to overcome defects derived from NFIB or MLL1 knockdown in C3H10T1/2 MSCs (Figs. 4 and S5). Furthermore, ChIP and ChIP-seq analysis confirmed selective bindings of the NFIB–MLL1 complex to both *Dlx5* and *Cebpa* even prior to lineage-specific differentiation signals in C3H10T1/2 MSCs (Figs. 2 and 5).

In addition to a GO term associated with osteoblast differentiation, GO analysis of 52 direct targets of the NFIB–MLL1 complex showed a strong association with GO terms, such as neuron development (GO:0048666), neurogenesis (GO:0022008), and brain development (GO:0007420) (Fig. 5*C*), raising a possibility that the NFIB–MLL1 complex might play roles in other types of multipotent stem cells such as NSCs. Interestingly, it has been reported that NFIB is highly expressed in NSCs (46), and MLL1 has been implicated in the regulation of a variety of stem cells, including postnatal NSCs and muscle satellite cells (40, 41). On the other hand, RNA-seq analyses demonstrated distinguishable changes in the transcriptome of NFIB or MLL1 knockdown MSCs depending on the lineage-specific differentiation stimuli, supporting the idea that the role of the NFIB–MLL1 complex is to maintain the stemness and plasticity of MSCs (Fig. 7).

**Figure 8:**
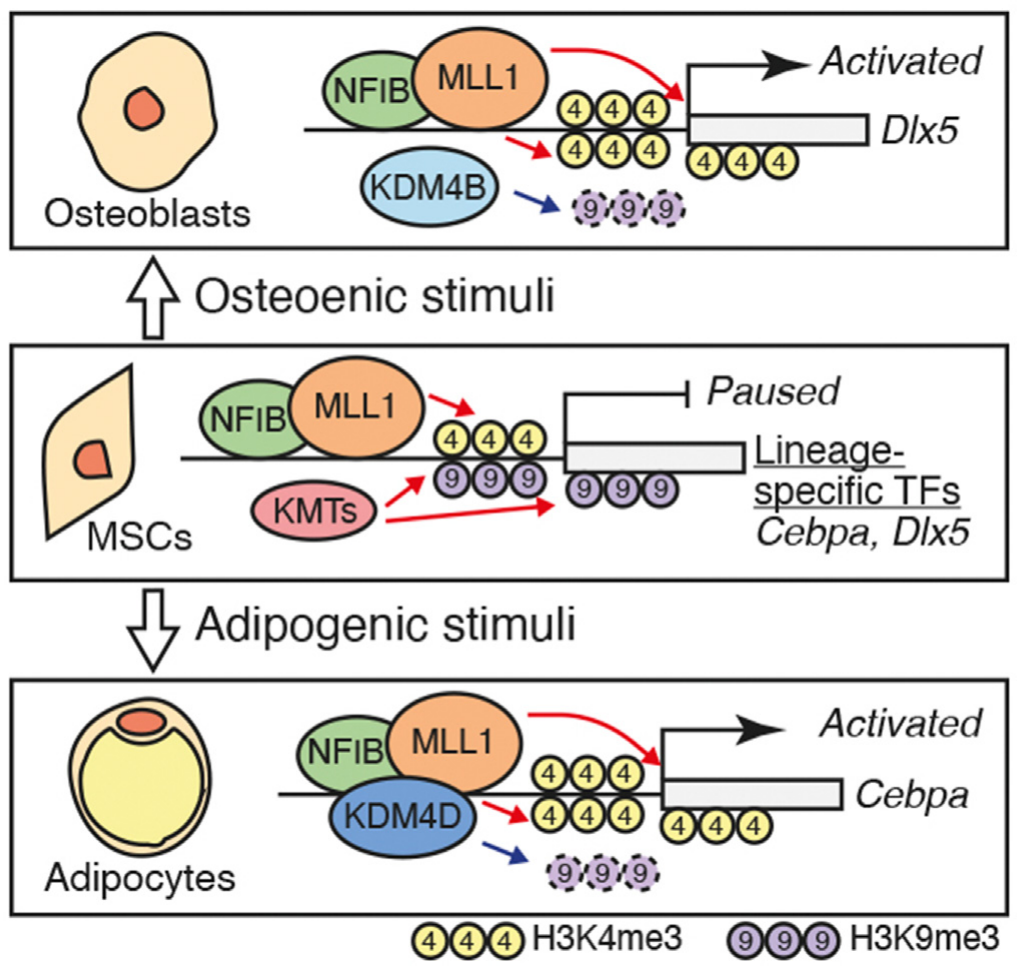
**A proposed model for the roles of the NFIB/MLL1 complex in the multilineage differentiation potential of C3H10T1/2 MSCs.** MSC, mesenchymal stem cell.

Considering that the depletion of NFIB or knockdown of MLL1 leads to a decreased level of H3K4me3 at the *Dlx5* and *Cebpa* promoters, the functions of the NFIB–MLL1 complex are likely related to the establishment of the chromatin landscape in uncommitted MSCs. Of particular interest, ChIP and ChIP-seq analysis revealed decreases in H3K4me3 accompanied by increases in repressive histone mark H3K9me3 at regions of *Dlx5* and *Cebpa*. These data support a previous report suggesting the bivalent chromatin domain comprised of H3K4me3 and H3K9me3 at the adipogenic regulator (61). In our proposed model, we speculate that genes critical for the initiation of lineage-specific differentiation, such as *Dlx5* and *Cebpa*, are kept at a ‘paused for response’ state by active H3K4me3 and repressive H3K9me3 until the relevant environmental cues in uncommitted C3H10T1/2 MSCs, in which the NFIB–MLL1 complex and SETDB1 histone lysine methylase could play key roles in establishing the bivalent chromatin domain (Fig. 8). Upon the differentiation signal, lineage-specific differentiation could occur either by the controlled displacement of SETDB1 or reversal of the H3K9me3 by the lineage-specific KDM at the developmental genes. The model supports the latter case because it has been shown that H3K9me3-specific KDMs play pivotal roles in lineage-specific differentiation by breaking the balance between the active and repressive histone marks at essential regulator genes (32, 44).

Lastly, our interpretation of the roles of the NFIB–MLL1 complex in the maintenance of MSCs is built on the results obtained from the experiments using cell lines derived from C3H10T1/2 MSCs. C3H10T1/2 is a MSC line derived from mouse embryo (62) and has been widely utilized as an *in vitro* model for MSCs differentiation, especially for its trilineage differentiation potential (63, 64, 65). In addition, dynamic changes in histone modifications and chromatin structure during lineage-specific differentiation are relatively well established in C3H10T1/2 MSCs (61, 66, 67). On the other hand, the possibility remains that MSCs maintenance by the NFIB–MLL1 complex might be limited to a specific cell line, C3H10T1/2 MSCs. Further studies using other MSCs, such as BM-MSCs, and other multipotent stem cells would confirm the roles of the NFIB–MLL1 complex in stem cell populations. Moreover, while functional redundancy among the NFI family of proteins remain as a possibility, it would be interesting to determine if defects observed from cells depleted of NFIB or MLL1 could be recapitulated *in vivo*.

In conclusion, the current study provides a molecular mechanism through which the stemness and plasticity of MSCs are maintained. Although it is widely accepted that the developmental genes are marked by both active and repressive histone modifications in various stem cells, these findings provide evidence that regulating the bivalent chromatin domain is critical for maintaining the MSC stemness and lineage-specific differentiation. Finally, given the critical roles of the NFIB–MLL1 complex in the stemness and plasticity of MSCs as well as in osteogenic differentiation, it is tempting to speculate that the NFIB–MLL1 complex might be able to serve as a potential therapeutic target for MSCs-related diseases such as osteoporosis (4, 5).

## Experimental procedures

### Plasmid DNAs, cell culture, and retroviral infection

Plasmids encoding FLAG-tagged NFIB and Myc-tagged Dlx5 have been described (44, 68). The plasmid containing complementary DNA for constitutively active β-catenin (S37A) was kindly provided by Dr E. H. Cho (University of Seoul). Mouse C3H10T1/2 MSCs and human Phoenix ecotropic packaging cells were maintained in Dulbecco’s modified Eagle’s medium (WelGENE) supplemented with 10% fetal bovine serum (WelGENE) and penicillin-streptomycin-glutamine solution (HyClone) in a humidified atmosphere containing 5% CO_2_ at 37 °C. The establishment of mouse C3H10T1/2 MSCs expressing shRNA that targets NFIB (shNFIB) or MLL1 (shMLL1) is described elsewhere (44). For retroviral gene transfer, replication-defective retroviruses were produced from Phoenix ecotropic cells transfected with relevant retroviral plasmids. The transfected cells were selected with 5 μg/ml puromycin (Sigma) for 24 h and then replenished with fresh medium without puromycin. The viral supernatants were collected after 24 h, filtered through a 0.45 μm syringe filter, and added to the cells of interest. To enhance infectivity, 8 μg/ml of polybrene (Sigma) was added to viral supernatants.

### *In vitro* osteogenic differentiation

*In vitro* osteogenic differentiation was induced by incubating cells in an osteogenic medium containing 20 mM β-glycerophosphate (Sigma), 100 μg/ml ascorbic acid 2-phosphate (Sigma), and 0.1 μM dexamethasone (Sigma). The osteogenic medium was changed every 2 days over 21 days, and differentiation was assessed by calcium deposits stained with ARS (Sigma). The stained calcium deposits were monitored using an Olympus BX50 microscope. All images were scanned with an Epson Perfection V500 scanner and processed using Adobe Photoshop CC 2022.

### Activation of the Wnt/BMP signaling pathways

The Wnt-signaling pathway was activated by incubating the cells with 25 mM lithium chloride (Sigma) or by the exogenous expression of constitutively active β-catenin (S37A). For BMP-signaling pathway activation, cells were cultured in a medium containing 100 ng/ml of recombinant human/mouse BMP2 (rhBMP2, R&D systems). The level of endogenous *Ccnd1* mRNA was monitored to evaluate the activation of the Wnt-signaling pathway. In addition, activation of the signaling pathways during osteogenic differentiation was assessed by increased calcium deposition and the expression of osteogenic marker genes as described previously (55, 56).

### Immunoblotting and RT-qPCR

The proteins of interest were analyzed by preparing whole cell lysates using RIPA buffer (10 mM Tris–HCl pH 7.5, 1 mM EDTA pH 8.0, 150 mM NaCl, 1% Triton X-100, 1% sodium deoxycholate, and 0.1% SDS) supplemented with protease inhibitor cocktail (Roche). After sonication using a VCX130 ultrasonic processor (SONICS, 20% amplitude for 20 s), the sonicated lysates were clarified by centrifugation at 4 °C for 20 min at 13,000*g*. The clarified supernatants were resolved by SDS-PAGE and analyzed by immunoblotting using an ECL plus Western Blotting Detection System (Cytiva) according to the manufacturer’s instructions. Table S1 lists the antibodies used for immunoblotting. For RT-qPCR analysis, the total RNAs were prepared using RNeasy plus mini kit (Qiagen), and complementary DNAs were synthesized using the GoScript reverse transcription system (Promega) according to the manufacturer’s instructions. Quantitative PCR was carried out on a QuantStudio 1 real-time PCR system (Thermo Fisher Scientific) with SYBR Green I (Invitrogen) and i-StarTaq DNA polymerase (Intron). The relative mRNA levels were calculated using the standard curve method and normalized to *Gapdh* mRNA. Table S2 lists the primers used for RT-qPCR.

### ChIP-qPCR assays

ChIP assays were performed as described previously with modifications (37). Briefly, the chromatins were prepared from cells fixed with 1% formaldehyde (Sigma) and sheared into fragments (200–700 bps) using an ultrasonic processor (at 45 W for 20 × 10 s pulses). The sonicated chromatins (50–100 μg) were precleared at 4 °C for 2 h and incubated with relevant antibodies. After immunoprecipitation, the recovered ChIP DNAs were analyzed by quantitative PCR on a QuantStudio 1 Real-Time PCR system with SYBR Green I and i-StarTaq DNA polymerase. For quantification, the percentage input (% input) was calculated using a standard curve method and normalized to IgG % input value. Tables S1 and S3 list the antibodies and primers used in the ChIP assays, respectively.

### RNA-seq and data processing

For RNA-seq, libraries were prepared from the total RNAs using TruSeq RNA-seq kit (Illumina), quantified using an Agilent Technologies 2100 Bioanalyzer (Agilent Technologies), and then sequenced on Illumina Nova-Seq 6000 platform (Illumina) with paired-end 100 bp read length to obtain at least 60 million reads per sample. The raw reads were subjected to basic quality control using FastQC version 0.11.9, and the adapter sequence was trimmed by Trimmomatic version 0.39 (69, 70). The resulting reads were mapped to the mouse genome assembly GRCm38 (mm10) using HISAT2 version 2.2.1 (71). The average read quality corresponding to each sequence alignment map (SAM) file is listed in Table S4. Reads with mapping quality score >30 were selected and converted to binary alignment map (BAM) files using SAMtools version 1.12 (72). After removing the PCR duplicates, sorted transcripts were assigned to the gene and counted using htseq-count version 0.11.1 (73).

### RNA-seq analysis

For RNA-seq analysis, the read counts obtained from data processing were first normalized using the trimmed mean of M-values method. The DEGs were then identified using R package edgeR version 3.38 (74). Significant DEGs were identified as those with a *p* value of <0.05 with Benjamini-Hochberg multiple testing correction at a 5% FDR and a fold change of >2. GO analysis was conducted using DAVID bioinformatics resources 6.8, and a significant biological process GO term was obtained by applying the thresholds of *p* < 0.05 (75). For cluster analysis, the gene clusters were determined using hclust and edgeR from the R package and visualized with the R package pheatmap. All charts were generated with the ggplot2 package from R software (https://ggplot2.tidyverse.org) and modified using Adobe Illustrator 2022.

### ChIP-seq and data processing

For ChIP-seq, sonicated chromatins (300–400 μg) were precleared with protein A/G and immunoprecipitated with relevant antibodies (Table S1). The integrity of the purified ChIP DNA was assessed using an Agilent Bioanalyzer 2100 (Agilent Technologies), and libraries were constructed using a TruSeq ChIP library preparation kit (Illumina). The prepared libraries were then sequenced on an Illumina Hi-Seq 2500 platform (Illumina), which generated approximately 2 to 3 GB of raw data with 100 bp paired-end read length. ChIP-seq reads were subjected to basic quality control using FastQC, and the adapter sequence was trimmed by Trimmomatic. The resulting reads were aligned to the mm10 reference genome using Bowtie2 version 2.4.2 (76). Table S4 lists the average read quality corresponding to each SAM file. The aligned reads with mapping quality score >30 were converted into BAM format using SAMtools. For peak calling analysis and distribution analysis, browser-extensible data files were generated from sorted BAM files using the bamToBed function of Bedtools version 2.30.0 (77).

### ChIP-seq analysis

The bamCoverage of deepTools package version 3.5.1 was used to generate ChIP-seq coverage tracks. Read coverage was normalized using read per genome coverage, and the coverage tracks were visualized using Integrative Genomic Viewer (78, 79). The average signal profiles around TSSs for NFIB, MLL1, and H3K4me3 were generated using ngs.plot.R version 2.61 (80). The NFIB, MLL1, and H3K4me3 peaks were assigned using SICER2 version 1.0.3 (81). Differential peaks analysis was performed using the sicer-df function of SICER2. The peaks were annotated using the R package ChIPseeker, defining the promoter region ±3 kb from the TSSs (82). GO term analysis was conducted using DAVID bioinformatics resources or enrichGO from the clusterProfiler R package (83).

### Statistical analysis

At least three independent experiments were performed as indicated in the figure legends and are presented as the means ± SD or ± SEM. Statistical analyses were conducted using GraphPad Prism software version 9 (https://www.graphpad.com). The normality of data was assessed using a Shapiro–Wilk test. For two groups, normally distributed data were analyzed using a two-sided *t* test, while skewed data were analyzed using a Mann-Whitney test. One-way or two-way ANOVA with a correction for multiple comparisons was performed for three or more groups. Statistical significance was defined as *p* <0.05.

## Data availability

All of the data are contained in the article and Supporting information. The data that support the findings of this study are available from the corresponding author upon reasonable request. All RNA-seq and ChIP-seq data are available at the GEO under SuperSeries number GSE227538.

## Supporting information

This article contains supporting information (20, 44, 68, 84, 85, 86, 87, 88, 89, 90, 91, 92, 93).

## Conflict of interest

The authors declare that they have no conflicts of interest with the contents of this article.
