# Supplementary material for: NFIB–MLL1 complex is required for the stemness and Dlx5-dependent osteogenic differentiation of C3H10T1/2 mesenchymal stem cells
Source: J Biol Chem. 2023 Aug 24;299(10):105193. doi: 10.1016/j.jbc.2023.105193 (PMC10519831; doi:10.1016/j.jbc.2023.105193)
Supplement: Supporting information [file mmc1.pdf]

Supporting Information

The NFIB/MLL1 complex is required for the stemness and Dlx5-dependent osteogenic differentiation of C3H10T1/2 mesenchymal stem cells

Janghyun Choi[1,*] and Hansol Lee[1,*]

[1]Department of Biological Sciences, College of Natural Science, Inha University, 100 Inha-ro, Michuhol-gu, Incheon, Korea, 22212

***Corresponding authors.**

hlee@inha.ac.kr (H. Lee) and jchoi@inha.ac.kr (J. Choi)

List of contents:

Supporting Information Figure S1 – S7 (pages S-2 to S-8)

Supporting Information Figure Legends (pages S-9 to S-12)

Supporting Information Table S1 – S4 (pages S-13 to S-16)

Supporting Information Data S1 (page S-16 and spreadsheet file)

Supporting Information Materials and Methods (page S-17)

Supporting Information Figures

Figure S1

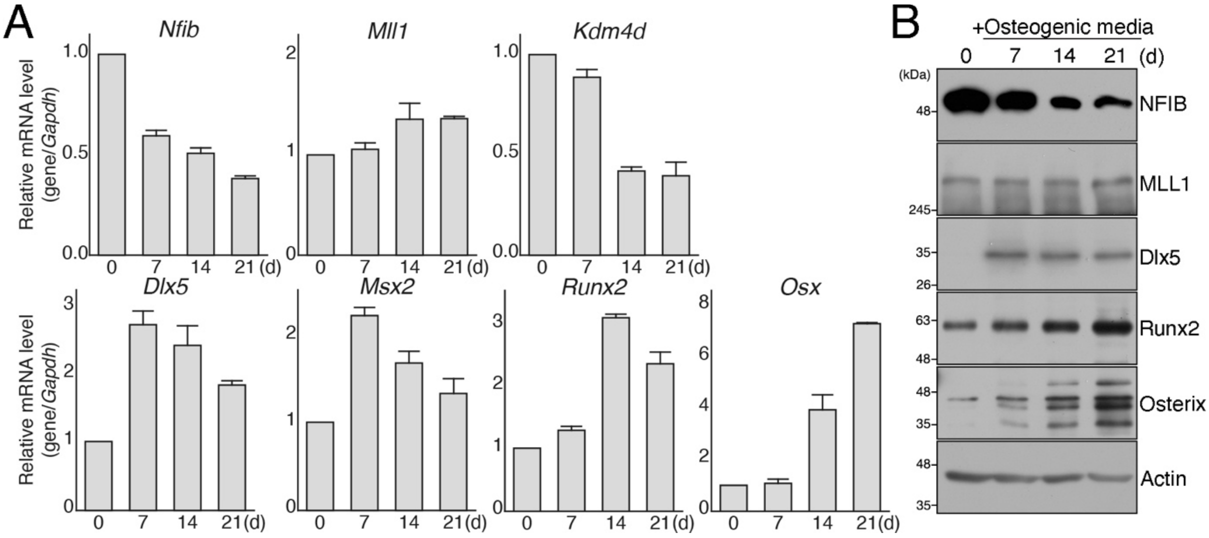

Figure S2

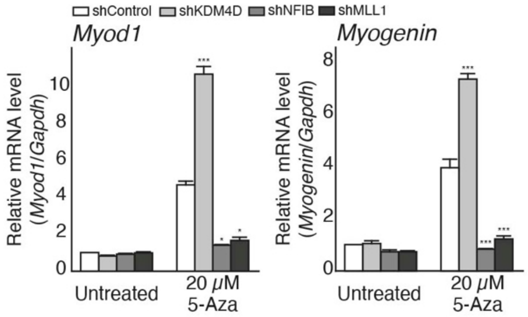
shControl
shKDM4D
shNFIB
shMLL1
Myod1
Relative mRNA level (Myod1/Gapdh)
0
2
4
6
8
10
Untreated
20 μM 5-Aza
Myogenin
Relative mRNA level (Myogenin/Gapdh)
0
2
4
6
8
Untreated
20 μM 5-Aza

Figure S3

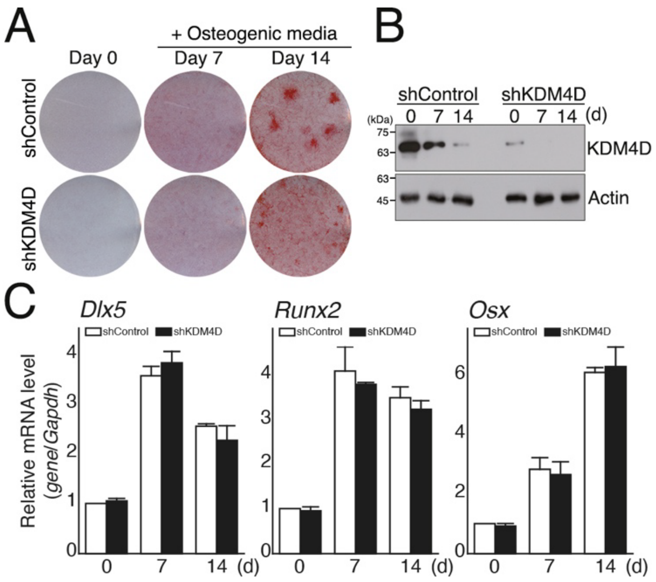
A
+ Osteogenic media
Day 0
Day 7
Day 14
shControl
shKDM4D
B
shControl
shKDM4D
(kDa)
0 7 14
0 7 14 (d)
75
63
KDM4D
63
45
Actin
C
Dlx5
Runx2
Osx
shControl
shKDM4D
Relative mRNA level
(gene/Gapdh)
0 7 14 (d)

Figure S4

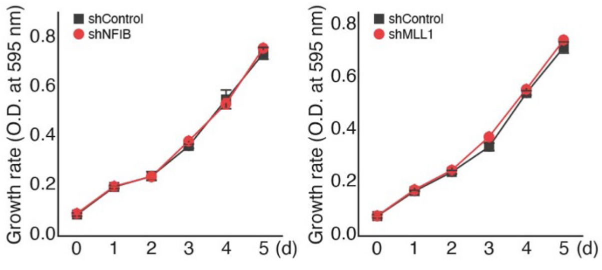
shControl
shNFIB
Growth rate (O.D. at 595 nm)
0.0
0.2
0.4
0.6
0.8
0
1
2
3
4
5 (d)
shControl
shMLL1
Growth rate (O.D. at 595 nm)
0.0
0.2
0.4
0.6
0.8
0
1
2
3
4
5 (d)

Figure S5

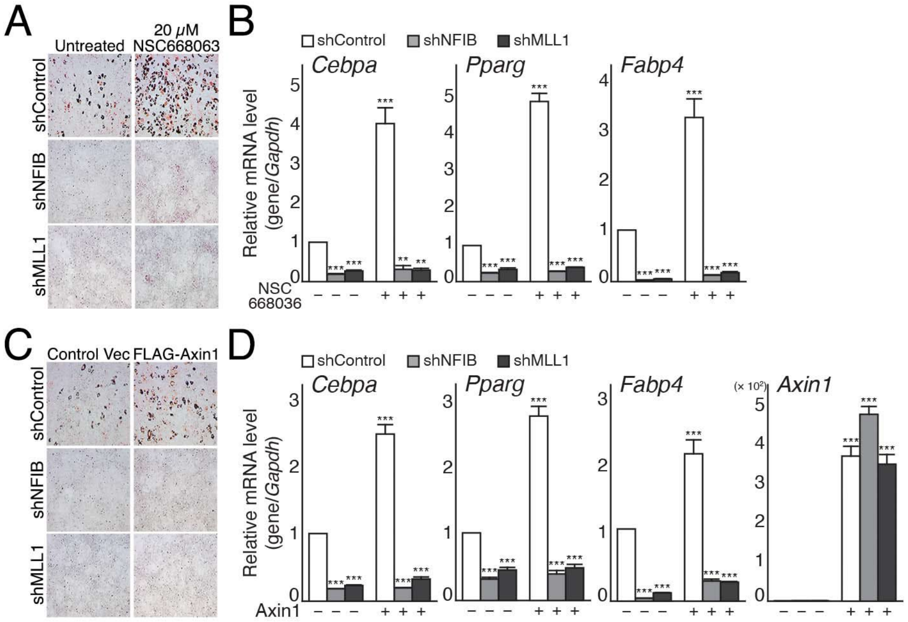
A
Untreated
20 μM NSC668063
shControl
shNFIB
shMLL1
B
shControl
shNFIB
shMLL1
Cebpa
Pparg
Fabp4
Relative mRNA level (gene/Gapdh)
NSC 668036
C
Control Vec
FLAG-Axin1
D
Axin1
(× 10²)
Axin1

Figure S6

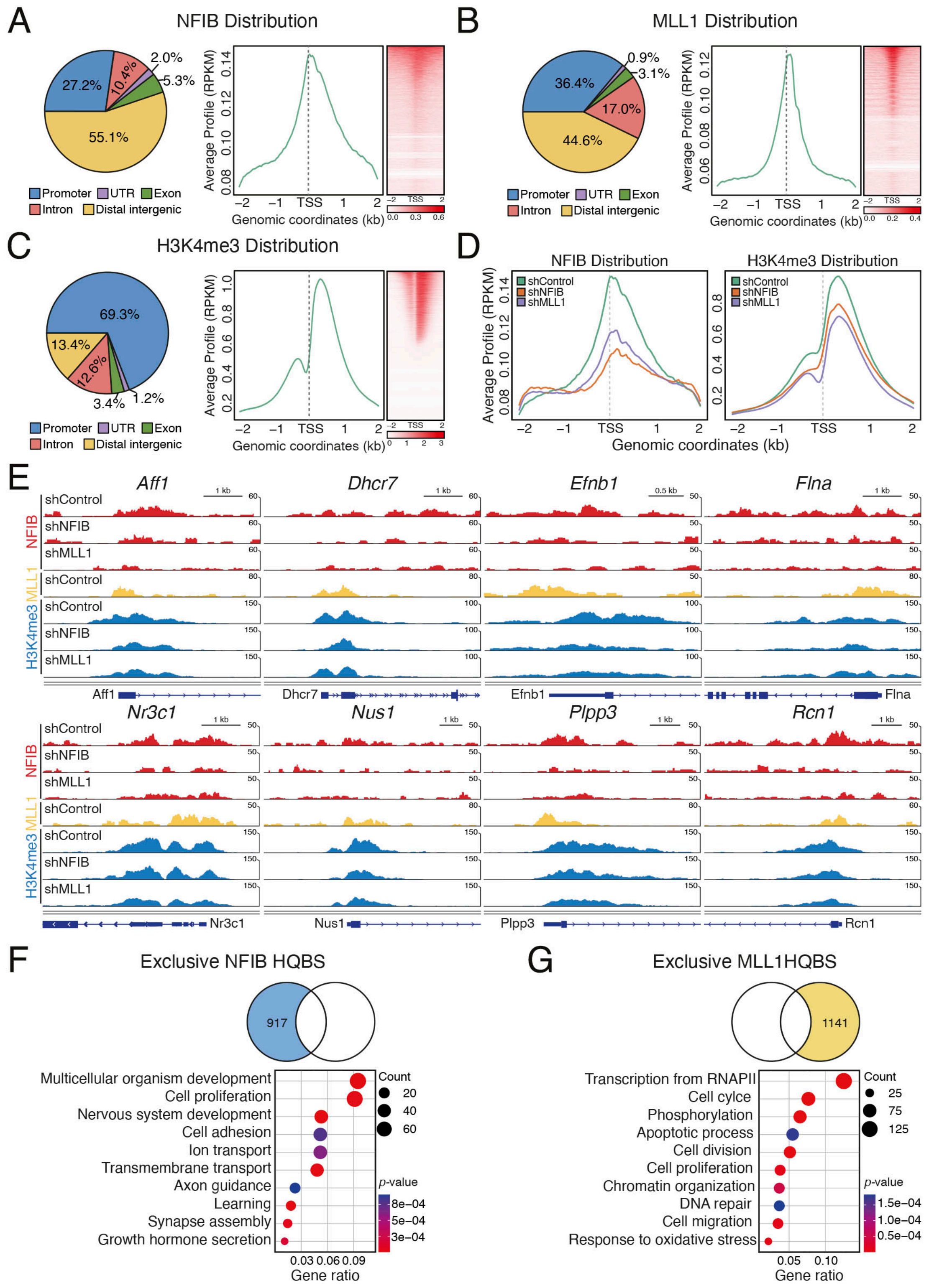

A
NFIB Distribution
27.2%
10.4%
2.0%
5.3%
55.1%
Promoter
UTR
Exon
Intron
Distal intergenic
Average Profile (RPKM)
Genomic coordinates (kb)
B
MLL1 Distribution
36.4%
0.9%
3.1%
17.0%
44.6%
C
H3K4me3 Distribution
69.3%
13.4%
12.6%
3.4%
1.2%
D
NFIB Distribution
H3K4me3 Distribution
shControl
shNFIB
shMLL1
E
Aff1
Dhcr7
Efnb1
Flna
Nr3c1
Nus1
Plpp3
Rcn1
NFIB
MLL1
H3K4me3
F
Exclusive NFIB HQBS
917
Multicellular organism development
Cell proliferation
Nervous system development
Cell adhesion
Ion transport
Transmembrane transport
Axon guidance
Learning
Synapse assembly
Growth hormone secretion
Count
p-value
Gene ratio
G
Exclusive MLL1HQBS
1141
Transcription from RNAPII
Cell cylce
Phosphorylation
Apoptotic process
Cell division
Cell proliferation
Chromatin organization
DNA repair
Cell migration
Response to oxidative stress

Figure S7

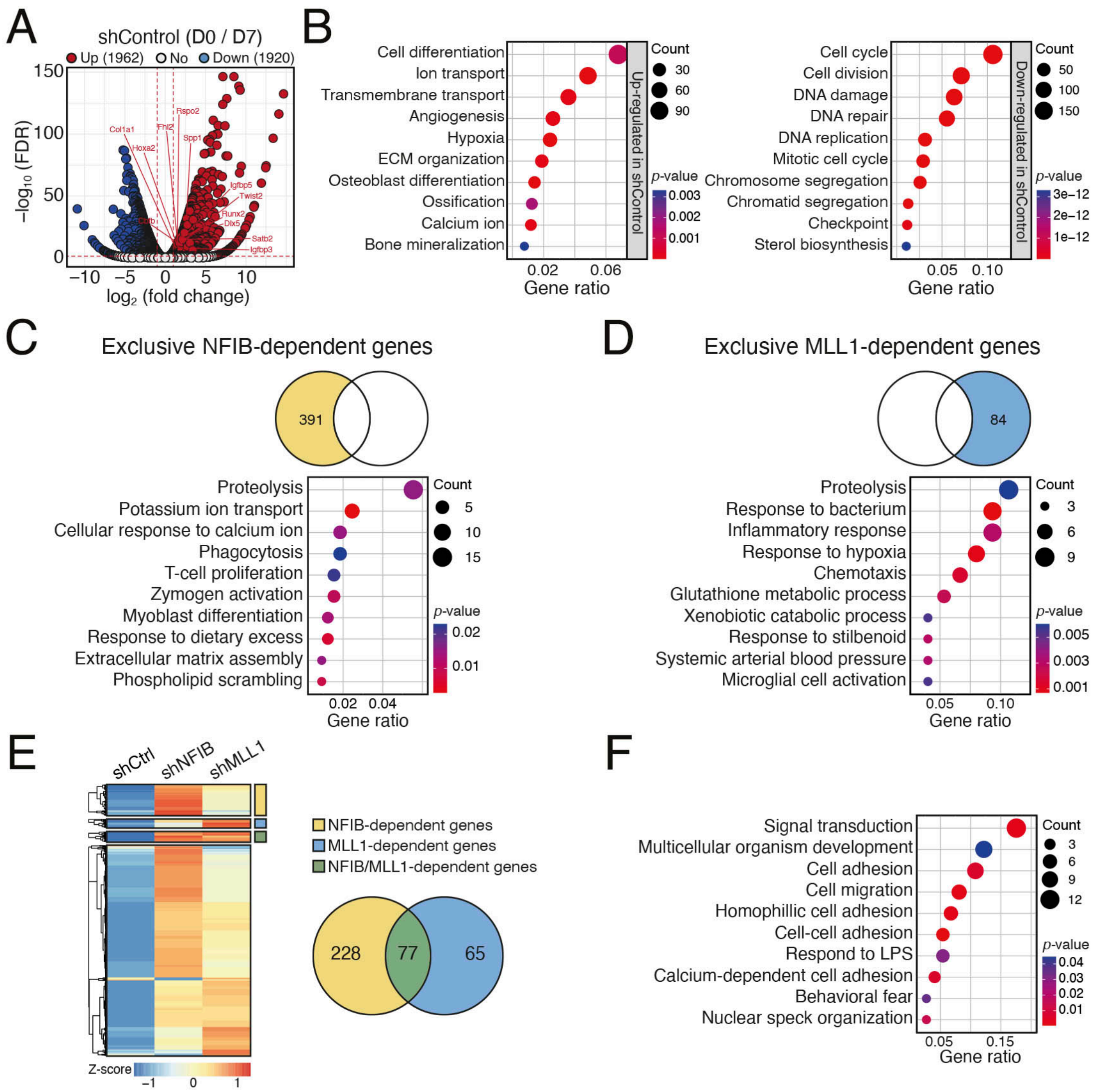
A
shControl (D0 / D7)
Up (1962) No Down (1920)
−log10 (FDR)
log2 (fold change)
B
Cell differentiation
Ion transport
Transmembrane transport
Angiogenesis
Hypoxia
ECM organization
Osteoblast differentiation
Ossification
Calcium ion
Bone mineralization
Up-regulated in shControl
Gene ratio
Count
p-value
Cell cycle
Cell division
DNA damage
DNA repair
DNA replication
Mitotic cell cycle
Chromosome segregation
Chromatid segregation
Checkpoint
Sterol biosynthesis
Down-regulated in shControl
C
Exclusive NFIB-dependent genes
391
Proteolysis
Potassium ion transport
Cellular response to calcium ion
Phagocytosis
T-cell proliferation
Zymogen activation
Myoblast differentiation
Response to dietary excess
Extracellular matrix assembly
Phospholipid scrambling
D
Exclusive MLL1-dependent genes
84
Proteolysis
Response to bacterium
Inflammatory response
Response to hypoxia
Chemotaxis
Glutathione metabolic process
Xenobiotic catabolic process
Response to stilbenoid
Systemic arterial blood pressure
Microglial cell activation
E
shCtrl
shNFIB
shMLL1
Z-score
NFIB-dependent genes
MLL1-dependent genes
NFIB/MLL1-dependent genes
228
77
65
F
Signal transduction
Multicellular organism development
Cell adhesion
Cell migration
Homophillic cell adhesion
Cell-cell adhesion
Respond to LPS
Calcium-dependent cell adhesion
Behavioral fear
Nuclear speck organization
Gene ratio

Supporting Information Figure legends

Figure S1. Expression of NFIB and MLL1 during osteogenic differentiation of C3H10T1/2 MSCs. Related to Figure 1. C3H10T1/2 cells were grown to confluence and further incubated in osteogenic medium containing 20 mM β-glycerophosphate, 100 μg/mL ascorbic acid 2-phosphate, and 0.1 μM dexamethasone up to 21 days. ***A***, RT-qPCR analysis of *Nfib*, *Mll1*, *Kdm4d* and key osteogenic regulator genes such as *Dlx5*, *Msx2*, *Runx2*, and *Osx*. For each gene, relative mRNA values were calculated as relative to the value at day 0 following normalization to *Gapdh* mRNA. Data were presented as mean ± SD (n = 3 independent experiments). ***B***, immunoblot results of NFIB, MLL1, and key osteogenic regulators. Actin was used as a loading control. Whole cell extracts (WCE) were prepared at indicated time points (0, 7, 14, and 21 days). All images were from a single round of experiments.

Figure S2. NFIB and MLL1 are required for the myogenic differentiation of C3H10T1/2 MSCs. Related to Figure 1. shControl, shNFIB, shMLL1, and shKDM4D cells were incubated in the presence or absence of 20 μM of 5-Aza for 14 days. Shown are the RT-qPCR results of *Myod1* (*left*) and *Myogenin* (*right*) in shControl (white), shKDM4D (light gray), shNFIB (gray), and shMLL1 (dark gray) cells. Relative mRNA levels were calculated as relative to untreated shControl cells following normalization to *Gapdh*. Data were analyzed using two-way ANOVA with Dunnett's post hoc test and presented as mean ± SD (n = 3 independent experiments); *$p < 0.05$, **$p < 0.01$, ***$p < 0.001$ versus shControl cells on day 14.

Figure S3. Depletion of KDM4D does not affect osteogenic differentiation of C3H10T1/2 MSCs. Related to Figure 1. shControl and KDM4D-depleted C3H10T1/2 (shKDM4D) cells were cultured in osteogenic medium for 14 days. ***A***, ARS staining results of shControl (*upper*) and shKDM4D (*lower*) cells. Cells were fixed and stained before (Day 0) and after (Day 7 and 14) osteogenic induction. Shown are representative data of 3 independent experiments and images were from a single round of experiments. ***B***, immunoblot results of KDM4D and Actin in shControl (*left*) and shKDM4D (*right*) cells at indicated time points (0, 7, and 14 days). Actin was used as a loading control. Images were representative of at least 3 independent experiments. ***C***, RT-qPCR analysis *Dlx5*, *Runx2*, and *Osx* in

shControl (white) and shKDM4D (black) cells. For each gene, relative mRNA levels were calculated as relative to shControl cells (Day 0) following normalization to *Gapdh*. Data were presented as mean ± SD (n = 3 independent experiments).

Figure S4. Depletion of NFIB or MLL1 has no significant effect on cell proliferation. Related to Figure 1. shControl, shNFIB, and shMLL1 cells were seeded and cultured for 5 days. Cell proliferation was assessed using NBB staining every 24 h for 5 days. Shown on the left are relative cell proliferations of shControl (black square) and shNFIB (red circle) cells. Shown on the right are relative cell proliferations of shControl (black square) and shMLL1 (red circle) cells. Data were presented as mean ± SD (n = 3 independent experiments).

Figure S5. Inhibition of Wnt-signaling does not rescue adipogenic defects in shNFIB and shMLL1 cells. Related to Figure 4. ***A and B***, shControl, shNFIB, and shMLL1 cells were treated with 20 μM of NSC668063 for 24 h and induced to differentiate into adipocytes by MDI hormonal induction. ***A***, Oil red O (ORO) staining results of untreated cells (*left*) and NSC668063-treated cells (*right*) after 8 days of incubation under adipogenic stimuli; shControl (*top*), shNFIB (*middle*), and shMLL1 (*bottom*). All images were obtained from a single round of experiments. Scale bar, 200 μm. ***B***, RT-qPCR results of *Cebpa*, *Pparg*, and *Fabp4* in shControl (white), shNFIB (grey), and shMLL1 (dark grey) cells. For each gene, relative mRNA levels were calculated as relative to untreated (−) shControl cells following normalization to *Gapdh*. ***C and D***, shControl, shNFIB, and shMLL1 cells were infected with retrovirus expressing FLAG-tagged Axin1 or control vector followed by induction of adipogenic differentiation for 8 days. ***C***, ORO staining results of shControl (*top*), shNFIB (*middle*), and shMLL1 (*bottom*) cells infected with empty retroviral vector (*left*) or retrovirus expressing FLAG-Axin1 (*right*). All images were from a single round of experiments. Scale bar, 200 μm. ***D***, RT-qPCR results of *Cebpa*, *Pparg*, *Fabp4*, and *Axin1* in shControl (white), shNFIB (grey), and shMLL1 (dark grey) cells. For each gene, relative mRNA levels were calculated as relative to untreated (−) shControl cells following normalization to *Gapdh*. For Figure S5B and S5D, data were analyzed using two-way ANOVA with

Dunnett's post hoc test and presented with mean ± SD (n = 3 independent experiments); $^{**}p < 0.01$, $^{***}p < 0.001$ versus untreated/empty vector-infected shControl cells (−).

Figure S6. Genome-wide identification of NFIB/MLL1 targets in C3H10T1/2 MSCs. Related to Figure 5. ***A* – *C***, shown on the left are pie charts depicting genomic distribution of (***A***) NFIB binding, (***B***) MLL1 binding, and (***C***) H3K4me3-enriched peaks (%) identified in undifferentiated C3H10T1/2 MSCs. Shown on the right are average ChIP-seq profiles of (***A***) NFIB, (***B***) MLL1, and (***C***) H3K4me3 across transcription start sites (TSS). ***D***, plots showing average ChIP-seq profiles of NFIB (*left*) and H3K4me3 (*right*) around TSS in shControl (green), shNFIB (red), and shMLL1 (purple) cells. ***E***, Interactive Genomics Viewer (IGV) genome browser snapshots of genes directly targeted by NFIB/MLL1 such as *Atf1*, *Dhcr7*, *Efnb1*, *Fina*, *Nr3c1*, *Nus1*, *Plpp3*, and *Rcn1* showing tracks of NFIB (red), MLL1 (yellow), and H3K4me3 (blue) ChIP-seq tag counts in undifferentiated shControl, shNFIB, and shMLL1 cells. ***F***, Venn diagram (*upper*) shows 917 targets (blue) that are exclusively NFIB HQBS but not MLL1 HQBS. Gene ontology (GO) classification (*lower*) showing the biological process (BP) terms of exclusively NFIB HQBS (n = 917). ***G***, Venn diagram (*upper*) shows 1,141 targets (yellow) that are exclusively MLL1 HQBS, but not NFIB HQBS. GO classification (*lower*) showing the BP terms of exclusively MLL1 HQBS (n = 1,141). For Figure S6F and S6G, the size of bubble indicates the number of genes. Color depth indicates significance ($p < 0.05$). The x-axis represents enrichment abundance (hit genes / populated genes) and the y-axis different GO terms.

Figure S7. The NFIB/MLL1 complex regulates lineage-specific changes in the transcriptome of C3H10T1/2 MSCs. Related to Figure 7. ***A***, volcano plot representing differentially expressed genes (DEGs) in response to osteogenic stimuli for 7 days in shControl cells. The red and blue circles represent the significantly upregulated and downregulated genes following osteogenic stimuli, respectively. The gray circle represent gene with no significant change in expression. ***B***, gene ontology (GO) classification showing the downregulated OAGs (*right*) in C3H10T1/2 MSCs. ***C***, Venn diagram (*upper*) shows 391 genes (yellow) that are exclusively NFIB-dependent, but not MLL1-dependent. Gene ontology (GO) classification (*lower*) showing the biological process (BP) terms of exclusively NFIB-

dependent genes (n = 391). ***D***, Venn diagram (*upper*) shows 84 genes (blue) that are exclusively MLL1-dependent, but not NFIB-dependent. Gene ontology (GO) classification (*lower*) showing the biological process (BP) terms of exclusively MLL1-dependent genes (n = 84). ***E***, shown on the left are hierarchical clustering results of 1,920 downregulated OAGs in shControl, shNFIB, and shMLL1 cells. 1,920 OAGs were differentially expressed genes with |log2 fold change| > 1 and FDR < 0.05 upon osteogenic stimuli in shControl cells. OAGs are clustered into 4 groups and manually curated based on NFIB and/or MLL1 dependency upon osteogenic stimuli: OAGs that failed to be downregulated only in shNFIB cells (NFIB-dependent genes, yellow), OAGs whose expressions were significantly increased in shMLL1 cells but not in shNFIB cells (MLL1-dependent genes, blue), OAGs showing NFIB- and MLL1-dependency (NFIB/MLL1-dependent genes, green), and OAGs with no significant dependency. The scale bar is based on the Z-score of log2 (CPM + 1). Shown on the right are Venn diagram showing 77 NFIB/MLL1-dependent OAGs (green) commonly presented in 305 NFIB-dependent downregulated OAGs and in 142 MLL1-dependent downregulated OAGs. ***F***, GO analysis of 77 downregulated OAGs exhibiting NFIB- and MLL1- dependent downregulated genes. For GO classifications in Figure S7B, S7C, S7D, and S7F, the size of bubble indicates the number of genes. Color depth indicates significance ($p < 0.05$). The x-axis represents enrichment abundance (hit genes / populated genes) and the y-axis different GO terms.

Supporting Information Tables

Table S1. Information on the antibodies used in this study.

**Antibody**	**Source**	**Identifier**	**Application**
NFIB	Abcam	ab186738;<br>RRID: AB_2782951	WB (1/2,000)
NFIB	Atlas antibodies	HPA003956<br>RRID: AB 1854424	ChIP (4 μg), ChIP-seq (8 μg)
KDM4D	Abcam	ab93694;<br>RRID: AB_10561782	WB (1/5,000)
MLL1	Bethyl laboratory	A300-086A;<br>RRID: AB_242510	WB (1/10,000), ChIP (4 μg), ChIP-seq (8 μg)
Dlx5	Cell signaling	ab109737;<br>RRID: AB_10862856	WB (1/5,000)
Runx2	Cell signaling	#12556;<br>RRID: AB_2732805	WB (1/2,000)
Osterix	Abcam	ab22552;<br>RRID: AB_2194492	WB (1/1,000)
Myc	(68)	N/A	WB (1/3,000)
FLAG	Sigma	F1804;<br>RRID: AB_262044	WB (1/3,000)
Actin	Sigma	A1978;<br>RRID: AB_476692	WB (1/20,000)
H3K4me3	Abcam	ab8580;<br>RRID: AB_306649	ChIP (2 μg), ChIP-seq (4 μg)
H3K9me3	Abcam	ab8898;<br>RRID: AB_306848	ChIP (4 μg)
Rabbit IgG	Vectashield	I-1000;<br>RRID: AB_2336355	ChIP (2 μg)
Mouse IgG-HRP	GE	NA-931V;<br>RRID: AB_772210	WB (1/10,000)
Rabbit IgG-HRP	Jackson ImmunoResearch	711-035-152;<br>RRID: AB_10015282	WB (1/10,000)

Table S2. Information on the primers used for RT-qPCR

Gene	Forward primer	Reverse primer	Source
Nfib	CCGGAATACCTGGAGTCG	GAAATGGCAACGGTGAGG	(44)
Mll1	GGCTCCAGCAAGAACAAAAG	TCACACCTGCAAATGAGAGC	(44)
Kdm4d	CTGGAAGAATCGCCTGTATG AGT	GTCTTGAATTGTTCCCAGGTG AC	(44)
Dlx5	CTGGCCGCTTTACAGAGAAG	CTGGTGACTGTGGCGAGTTA	(84)
Runx2	CAGACCAGCAGCACTCCATA	CAGCGTCAACACCATCATTC	(85)
Osx	AGCGACCACTTGAGCAAACA T	GCGGCTGATTGGCTTCTTCT	(86)
Msx2	AACACAAGACCAACCGGAA G	GCAGCCATTTTCAGCTTTTC	(84)
Ccnd1	GCGTACCCTGACACCAATCT	CAGGTCTCCTCCGTCTTGAG	(44)
Myod1	TCAGGTGCTTTGAGAGATCG AC	CGAAAGGACAGTTGGGAAGA GT	(87)
Myogenin	CACTGGAGTTCGGTCCCAA	TGTGGGCGTCTGTAGGGTC	(87)
Cebpa	CCCAGCGGTGCCTTGTGC	TCCTTCCCCCAGCCGTTAGTG	(44)
Pparg	GCCCTTTGGTGACTTTATGGA	GCAGCAGGTTGTCTTGGATG	(44)
Fabp4	TGCTGCAGCCTTTCTCACCT	AGCCCACTCCCACTTCTTTCA	(44)
Axin1	ACGGTACAACGAAGCAGAG AGCT	CGGATCTCCTTTGGCATTCGG TAA	(88)
Gapdh	GCCTCTCTTGCTCAGTGTCC	TGCGACTTCAACAGCAACTC	(44)

Table S3. Information on the primers used for ChIP-qPCR

Gene	Forward primer	Reverse primer	Source
Dlx5 promoter	CTCTTTAAGCAATGCTTTGTTG TGC	GGCGCAGCACAGCCTTGGTTA AATC	(20)
Dlx5 distal	TGACAGAGGCTTGGAGTCCTT	TCCTCTTCTGGTTCCCCTTT	(89)
Runx2 promoter	GGCTCCTTCAGCATTTGTGT	TGTCCTCTCCCTTTCCTTCC	(90)
Runx2 distal	AAGGAGTTTGCAAGCAGAGC	CAACTGAGTGTGTGGCGTTC	(90)
Osx promoter	GAAGCTCTGACAACTTGCCC	AAGGGAGAGGGAGGGAGAAT	(90)
Osx distal	ATACATATACATACATTCAC	TAGTTCAGGGTGGCAGTGTG	(90)

Table S4. Sequencing depth and mapping summary

Type	Sample	Knockdown	Condition	Mapped read (bp.)	Mapped rate (%)	Error rate (%)
RNA-seq	shCon_D0	WT	Day 0	78,328,254	96.5	0.18
	shCon_D7	WT	Day 7	76,719,113	96.2	0.18
	shMLL1_D0	MLL1	Day 0	76,159,689	95.9	0.18
	shMLL1_D7	MLL1	Day 7	58,834,569	96.2	0.18
	shNFIB_D0	NFIB	Day 0	82,329,046	97.3	0.19
	shNFIB_D7	NFIB	Day 7	76,016,682	96.2	0.19
ChIP-seq H3K4me3	IPT	WT	Day 0	23,447,395	86.9	0.92
	shCon_D0	WT	Day 0	24,316,397	98.4	1.32
	shCon_D7	WT	Day 7	35,380,501	98.4	1.25
	shMLL1_D0	MLL1	Day 0	26,062,736	98.2	1.77
	shMLL1_D7	MLL1	Day 7	30,448,145	98.2	1.89
	shNFIB_D0	NFIB	Day 0	26,049,077	98.2	1.76
	shNFIB_D7	NFIB	Day 7	31,277,100	98.6	0.91
ChIP-seq MLL1	IPT	WT	Day 0	47,471,013	96.2	1.17
	shCon_D0	WT	Day 0	38,231,251	97.2	1.25
ChIP-seq NFIB	shCon_D0	WT	Day 0	47,268,717	96.2	1.16
	shCon_D7	WT	Day 7	48,340,390	97.9	1.25
	shMLL1_D0	MLL1	Day 0	48,289,345	97.9	0.93
	shMLL1_D7	MLL1	Day 7	48,490,709	98.8	1.15
	shNFIB_D0	NFIB	Day 0	47,271,801	95.5	1.1
	shNFIB_D7	NFIB	Day 7	48,058,304	97	1.49

Supporting Information Data

Data S1. 162 NFIB/MLL1-dependent osteogenesis-associated genes (OAGs).

Supporting Information Materials and Methods

Cell proliferation assay

Cell proliferation was assessed using the naphthol blue black (NBB) staining protocol as previously described (91). Briefly, cells were seeded at a density of 2 × 10^3 cells per well in 24-well plates and cultured in DMEM supplemented with 10% FBS for 5 days. At the indicated time points (0, 1, 2, 3, 4, and 5 days), cells were fixed with 4% paraformaldehyde (PFA, Wako) at room temperature for 20 min, followed by washing with PBS twice. The cells were then stained with 500 μL of NBB solution, containing 9% acetic acid (Merck) and 0.1 M sodium acetate (Merck), at room temperature for 30 min. After staining, the cells were washed three times with deionized water and eluted with 150 μL of 50 mM NaOH (Sigma). Cell proliferation was quantified by measuring the absorbance at 595 nm using an XFluor4 microplate reader, and the results were expressed as optical density at 595 nm (O.D. 595).

Wnt inhibition and *in vitro* differentiation

Inhibition of the Wnt signaling pathway was achieved by incubation of cells in the presence of 20 μM NSC668063 (Sigma) or by exogenous expression of FLAG-Axin1, as previously reported (92, 93). Adipogenic differentiation was induced by incubating confluent cells in medium containing 10 μg/mL insulin (Sigma), 1 μM dexamethasone (Sigma), 0.5 mM isobutyl methylxanthine (Sigma), and 10% FBS for two days. Cells were then fed with DMEM supplemented with 10% FBS and 10 μg/mL insulin for two days, after which they were fed every other day with DMEM containing 10% FBS. Differentiation was monitored by the appearance of lipid droplets in cells, and then confirmed by Oil Red O (ORO) staining. The stained lipid droplets were visualized using a Leica DM750M light microscope equipped with a Leica DFC295 camera. All images were taken using a 100 × magnification and processed using the Adobe Photoshop CC 2022 software. For myogenic differentiation, C3H10T1/2 MSCs were grown to full confluency in DMEM supplemented with 10% FBS, and differentiation was induced in myogenic medium containing 20 μM 5-Aza (Sigma) for 14 days. The differentiation was assessed by quantifying the mRNA expression levels of key myogenic regulatory factors (MRFs), including *Myod1* and *Myogenin*.
